# Global, regional, and national burden of chronic kidney disease due to diabetes mellitus type 2 from 1990 to 2021, with projections to 2036: a systematic analysis for the Global Burden of Disease Study 2021

**DOI:** 10.3389/fmed.2025.1531811

**Published:** 2025-02-17

**Authors:** Ruikang Hu, Zhifeng Zhao, Luze Xie, Zhenjie Ma, Wen Wu, Shuangxi Li

**Affiliations:** ^1^Medical School of Chinese People’s Liberation Army (PLA), Beijing, China; ^2^College of Basic Medicinal Science, The Naval Medical University, Shanghai, China; ^3^Department of Respiratory Digestive and Occupational Disease Treatment, Military Hospital of Chinese People’s Liberation Army, Hanzhong, China; ^4^Deparment of Nephrology, Changhai Hospital, The Navy Military Medical University, Shanghai, China

**Keywords:** disease burden, Global Burden of Disease Study, chronic kidney disease, diabetes mellitus type 2, disability-adjusted life years, age-standardized rates

## Abstract

**Background:**

Chronic kidney disease (CKD) due to type 2 diabetes mellitus (T2DM) has emerged as a significant global health burden, with rising incidence and prevalence rates observed over the past decades.

**Methods:**

We utilized the latest data from the Global Burden of Disease Study (GBD) 2021. Firstly, we reported the number of incidence, prevalence, deaths, and Disability-Adjusted Life Years (DALYs) attributed to CKD due to T2DM, accompanied by their respective Age-Standardized Rates (ASRs), for the year 2021. This analysis encompassed a global perspective and was further stratified by various subtypes. Moreover, we examined trends globally and within specified sub-types to investigate the temporal dynamics of the ASRs. We estimated the percentage change in ASRs, providing a quantitative measure of the rate of change in the burden over the study period. Moreover, we utilized the Bayesian age-period-cohort (BAPC) model to forecast the future burden.

**Results:**

Globally, the ASRs of CKD due to T2DM all have witnessed a notable rise except for age-standardized prevalence rate (ASPR). The trends observed in both sexes and nearly all age groups were found to be congruent with those of the overall population. The increase in disease burden being greatest in the middle and lower SDI regions. The predicted results showed that the ASRs would still increase from 2022 to 2036.

**Conclusion:**

This study highlights the critical importance of addressing the growing burden of T2DM-related CKD on global health. Effective prevention and management strategies, including improvements in diabetes care, renal health promotion, and access to healthcare services, are urgently needed to mitigate the future impact of T2DM-related CKD.

## 1 Introduction

Chronic kidney disease (CKD) due to type 2 diabetes mellitus (T2DM) represents a significant global health burden, affecting millions of individuals worldwide and contributing substantially to morbidity and mortality (1). The escalating prevalence of T2DM, coupled with its complex interplay with various risk factors, has led to a surge in CKD cases attributed to this metabolic disorder (2, 3). Understanding the global, regional, and national patterns of this burden, as well as projecting future trends, is crucial for the formulation of effective public health strategies and resource allocation.

Over the past few decades, the global landscape of T2DM and its complications has undergone substantial transformations. The rapid urbanization, sedentary lifestyles, and unhealthy dietary habits have contributed to the dramatic rise in T2DM incidence (4, 5). Consequently, the number of individuals developing CKD as a complication of T2DM has also soared. CKD due to T2DM is characterized by progressive renal function decline, often leading to end-stage renal disease (ESRD) requiring renal replacement therapy, such as dialysis or kidney transplantation (6, 7). This transition not only impacts individual quality of life but also places considerable economic strain on healthcare systems globally (8, 9).

Previous studies have documented the regional variations in the burden of CKD due to T2DM. For instance, high-income countries with aging populations and a longer history of diabetes management have observed a shift towards more complex diabetes-related complications, including CKD (10). Conversely, middle-and low-income countries are experiencing a double burden, with both an increasing incidence of T2DM and a high prevalence of communicable diseases (11, 12). These disparities highlight the need for tailored interventions that address the unique challenges faced by different regions.

The GBD Study has been a pivotal platform for quantifying the health burden attributable to various diseases and injuries worldwide. Providing an extensive analysis of global health loss. It offers up-to-date data on the distribution and burden of diseases and injuries, taking into account temporal changes, age, sex, geographical location, and sociodemographic factors (13). Previous iterations of the GBD Study have provided valuable insights into the burden of CKD in general, but there is a scarcity of comprehensive data specifically focusing on CKD due to T2DM (14, 15). This gap in knowledge necessitates a dedicated analysis to dissect the intricate relationship between T2DM and CKD, allowing for a more nuanced understanding of the disease dynamics.

In this study, we leverage the robust methodology of the GBD Study to estimate the incidence, prevalence, mortality, and disability-adjusted life years (DALYs) lost due to CKD caused by T2DM across different geographical regions and countries. By employing a systematic approach to data collection, analysis, and projection, we aim to fill the existing knowledge gap and inform policymakers and healthcare providers about the evolving burden of this condition.

Our analysis builds upon previous literature by incorporating the latest available data, applying advanced statistical models for estimation and projection, and adopting a consistent framework for comparing findings across different time points and geographical areas. This approach ensures that our results are both comprehensive and comparable, allowing for accurate benchmarking and the identification of trends over time (16–18).

Furthermore, we extend our analysis beyond the current burden to provide projections until 2036. These projections, based on demographic and epidemiological trends, are essential for planning and preparing healthcare systems to meet the anticipated demand for CKD care due to T2DM (19, 20). By anticipating future needs, we can facilitate the timely allocation of resources and the development of targeted interventions that aim to reduce the incidence and progression of CKD in T2DM patients.

## 2 Materials and methods

### 2.1 Study design and data sources

The Global Burden of Disease (GBD) 2021^1^ study provides comprehensive global health data, covering 371 diseases and injuries across 204 countries and territories, with age-sex-location-year specific estimates for 88 risk factors at global, regional, and national levels from 1990 through 2021 (13, 15). This systematic analysis was conducted as part of the GBD Study 2021, aiming to estimate the global, regional, and national burden of CKD due to T2DM from 1990 to 2021, with projections to 2036. Data sources of GBD database included published literature, vital registration systems, ESRD registries, and household surveys (21, 22). The GBD study framework was utilized to ensure the standardization and comparability of estimates across different populations and time points (23, 24).

### 2.2 Definition and classification of CKD

In GBD 2021, CKD is defined as a permanent loss of kidney function as indicated by estimated glomerular filtration rate (eGFR) and urinary albumin to creatinine ratio (ACR) (25). T2DM is defined as a metabolic disorder in which the body does not respond normally to insulin, causing chronic high blood sugar (glucose) levels, which over time leads to serious damage to the heart, blood vessels, eyes, kidneys, and nerves (26). Collaborator-provided sources that were either shared directly with us or were identified through searching the Global Health Data Exchange (GHDx) were reviewed for inclusion. For this study, CKD attributable to T2DM was identified based on a combination of ICD codes, keywords in text, and additional criteria specified in the GBD study methodology (12).

### 2.3 Statistical analysis

We reported the number of incidence, prevalence, deaths, and DALYs attributed to CKD due to T2DM. The age-standardized incidence rate (ASIR), age-standardized prevalence rate (ASPR), age-standardized deaths rate (ASDR) and age-standardized DALYs rate (ASDAR) were utilized as indicators of the disease burden for CKD due to T2DM. This analysis encompassed a global perspective and was further stratified by various subtypes, encompassing age groups, sex, Socio-demographic Index (SDI) regions, GBD regions, and individual countries. Moreover, we examined trends globally and within specified sub-types to investigate the temporal dynamics of the age-standardized rates (ASRs). We estimated the percentage change in ASRs, providing a quantitative measure of the rate of change in the burden over the study period.

The Bayesian age-period-cohort (BAPC) model was utilized to forecast the future burden of CKD due to T2DM. Bayesian inference treats uncertain parameters as random variables with specified prior distributions, assuming temporal effects exhibit similarity. To model this, a second-order random walk (RW2) is commonly employed, smoothing age, period, and cohort effects under the assumption that the second differences of all time effects follow independent, mean-zero normal distributions (27). The BAPC model utilizes an integrated nested Laplacian approximation to estimate the marginal posterior distribution, addressing issues related to mixing and convergence that are typically encountered with the Markov chain Monte Carlo sampling method used in traditional Bayesian approaches (28). This model effectively handles age-stratified cancer incidence and mortality rates, making it particularly valuable for projecting future trends amidst substantial demographic changes (29).

Throughout the analysis, statistical significance was determined at a *p*-value threshold of <0.05. For all computations and analyses, we leveraged the R software (version 4.2.3) to perform the database construction, collation, and rigorous statistical analysis.

## 3 Results

### 3.1 The disease burden of CKD due to T2DM in 2021

In 2021, the number of incidence cases of CKD due to diabetes mellitus type was 477273.1 (95% uncertainty intervals (UI): 401541.1, 565951.0) globally. The corresponding ASIR was 5.7 (95% UI: 4.8, 6.8) per 100,000 population. The number of prevalence cases was 107559954.8 (95% UI: 99170797.2, 115994731.7) globally. The corresponding age-standardized prevalence rate (ASPR) was 1259.6 (95% UI: 1,162, 1359.9) per 100,000 population. The number of deaths cases was 2012024.5 (95% UI: 1,857,800, 2154287.7) globally. The corresponding ASDR was 23.1 (95% UI: 21.4, 24.7) per 100,000 population. Moreover, the number of DALYs was 11,278,935 (95% UI: 9682785.2, 13103870.8) globally in 2021. The corresponding ASDAR was 131.1 (95% UI: 112.8, 152.5) per 100,000 population (Table 1).

**Table 1:** The trends of the chronic kidney disease due to diabetes mellitus type 2-related age-standardized incidence, prevalence, deaths, and DALYs rate in 2021 and from 1990 to 2021 globally.

	Deaths			Prevalence			Incidence			DALYs		
Location	Counts 2021 (95% UI)	Age standardized rate 2021 (95% UI)	Percentage change in ASRs from 1990 to 2021	Counts 2021 (95% UI)	Age standardized rate 2021 (95% UI)	Percentage change in ASRs from 1990 to 2021	Counts 2021 (95% UI)	Age standardized rate 2021 (95% UI)	Percentage change in ASRs from 1990 to 2021	Counts 2021 (95% UI)	Age standardized rate 2021 (95% UI)	Percentage change in ASRs from 1990 to 2021
Global	477273.1 (401541.1, 565,951)	5.7 (4.8, 6.8)	37.8 (19.2, 49.6)	107559954.8 (99170797.2, 115994731.7)	1259.6 (1,162, 1359.9)	−5.1 (−7.5, −3)	2012024.5 (1,857,800, 2154287.7)	23.1 (21.4, 24.7)	21 (15, 27.5)	11,278,935 (9682785.2, 13103870.8)	131.1 (112.8, 152.5)	24 (9.3, 33)
Low SDI	29491.3 (23284.4, 37040.8)	7.4 (5.9, 9.3)	2.8 (−10.1, 15.9)	8155173.7 (7,427,183, 9026802.9)	1269.4 (1,167, 1378.8)	−6.3 (−8.7, −4.4)	77,298 (69537.2, 84888.8)	15.1 (13.6, 16.6)	26.8 (20, 34.4)	761198.2 (622327.6, 942049.5)	160.9 (131.7, 199.6)	−3.3 (−13.7, 8)
Low-middle SDI	82399.1 (66442.9, 102844.3)	6.4 (5.2, 8)	31.2 (4.7, 51.6)	23752428.1 (21773064.6, 25995903.5)	1474.6 (1357.9, 1606.5)	−7.1 (−9.4, −4.9)	298098.8 (270253.9, 326985.4)	20.2 (18.3, 22.1)	33.6 (27.6, 40)	2202183.6 (1821393.4, 2694852.3)	155.3 (128.6, 189.4)	24.2 (3.4, 41)
Middle SDI	182159.5 (151834.7, 216847.2)	7.5 (6.2, 9)	11 (−9.4, 23.6)	36412910.7 (33,598,848, 39348242.6)	1331.6 (1232.1, 1433.5)	−7 (−9.5, −4.8)	635310.5 (582063.2, 685432.6)	23 (21.1, 24.6)	34 (25.1, 43.9)	4424358.6 (3759638.1, 5160625.7)	167.1 (141.3, 193.9)	5.8 (−10.7, 16.4)
High-middle SDI	71227.1 (57909.3, 86608.8)	3.7 (3, 4.4)	21.5 (0.6, 39.9)	21060240.7 (19314069.1, 22837224.8)	1,157 (1062.1, 1,253)	−9 (−11.9, −6.6)	404153.8 (369299.5, 435463.9)	20.1 (18.4, 21.6)	27.8 (20.7, 36.3)	1678678.7 (1404999.2, 2018747.1)	84.7 (71.1, 102.2)	8.3 (−7.3, 21.9)
High SDI	111564.7 (93260.1, 132403.9)	4.6 (3.9, 5.4)	95.4 (79.5, 113.5)	18097945.9 (16763917.4, 19359375.7)	997.1 (918.7, 1066.3)	−3.9 (−5.6, −2.2)	595270.9 (547942.9, 636879.1)	28.3 (26.2, 30.3)	10.8 (4.8, 17.4)	2202413.4 (1928997.7, 2486233.2)	102.6 (90, 114.5)	64.3 (53.9, 74.8)
Oceania	795.9 (636.2, 1,004)	13.6 (11.2, 17.3)	27.3 (−10.2, 80.5)	127251.7 (111037.3, 142276.6)	1337.2 (1193.9, 1478.5)	−4.3 (−7.6, −1.3)	1223.5 (1066.5, 1377.5)	15.5 (13.8, 17.2)	27.9 (16.6, 39.2)	22599.4 (18308.5, 27917.9)	309.8 (257.3, 383.9)	23.8 (−10.4, 71.1)
Central Europe	2761.1 (2138.6, 3486.9)	1.1 (0.9, 1.4)	5.4 (−4.8, 16.6)	1685179.9 (1564182.8, 1814448.7)	855.7 (792.6, 922.2)	−8.8 (−12.5, −6.3)	49402.8 (44902.9, 54054.3)	22.1 (20.2, 23.9)	68.2 (57.5, 81.2)	75975.3 (62114.3, 92514.6)	33.5 (27.3, 40.6)	−2 (−9, 5.6)
High-income Asia Pacific	23596.8 (17306.6, 30380.1)	3.6 (2.8, 4.5)	−18.3 (−25.2, −13)	4607840.5 (4243578.2, 4946981.8)	1275.4 (1168.2, 1377.4)	−11.8 (−13.6, −10)	133311.7 (121632.4, 145016.1)	29.3 (26.9, 31.7)	1.2 (−3.2, 6.7)	394459.4 (318657.4, 468493.2)	75.2 (62.2, 87.2)	−18.9 (−23.2, −15.1)
Eastern Europe	2797.6 (2151.7, 3595.9)	0.8 (0.6, 1)	142.7 (115.2, 168.5)	4315524.3 (3951875.5, 4696855.2)	1390.3 (1277.4, 1520.4)	−9.6 (−13.9, −6.5)	52339.2 (47044.3, 58222.3)	15.3 (13.8, 16.8)	76.7 (65.9, 86.3)	95,788 (76259.4, 118768.3)	26.7 (21.4, 33)	25.7 (13.4, 40)
Southeast Asia	58970.4 (48619.2, 70561.1)	10.4 (8.6, 12.5)	31.4 (5.6, 52.1)	12333536.2 (11263092.3, 13439668.8)	1739.3 (1595.7, 1883.9)	−3 (−5.5, −0.7)	152649.9 (138455.5, 167650.1)	22.3 (20.3, 24.3)	50.3 (40.5, 61.1)	1535393.1 (1287456.3, 1818898.3)	237.7 (199.8, 276.1)	23.5 (2.9, 40.5)
Central Asia	1,289 (978.6, 1669.6)	1.8 (1.3, 2.3)	177.9 (122.1, 241.4)	1315604.5 (1205400.2, 1431740.6)	1,494 (1381.4, 1617.5)	−4.5 (−7.8, −1.7)	14528.3 (12630.8, 16383.7)	16.4 (14.6, 18.2)	77.1 (67.2, 88.6)	54913.9 (43760.1, 67,787)	67.5 (54.1, 82.9)	52.9 (35.6, 72.5)
East Asia	115063.7 (91638.3, 141544.9)	5.8 (4.7, 7.2)	−16.6 (−35.7, 1.5)	21662039.1 (19882229.6, 23414195.4)	1054.1 (972.3, 1140.1)	−13.1 (−15.7, −10.9)	373610.9 (340685.9, 402345.5)	16.6 (15.3, 17.8)	8.3 (0.5, 17.9)	2697277.8 (2197710.8, 3240412.2)	125.6 (103.4, 149.7)	−20.9 (−35.6, −5.9)
Australasia	506.1 (367.1, 689.5)	0.8 (0.6, 1.1)	62.5 (42.3, 85.2)	378015.1 (344562.5, 414093.6)	768.6 (695, 843.2)	−7.1 (−12.3, −3.6)	16772.4 (14864.1, 18,754)	30.3 (27, 33.9)	13.7 (4.7, 22.2)	12981.3 (10124.5, 16242.5)	23.6 (18.3, 29.5)	29 (15.6, 44.9)
Southern Latin America	4602.8 (3602.9, 5792.2)	5 (3.9, 6.3)	−2.8 (−10.5, 4.9)	753904.9 (680382.9, 837547.9)	911.8 (819.8, 1021.1)	3 (−1, 6.6)	25927.7 (23175.9, 28694.4)	29.2 (26.2, 32.3)	33 (20.1, 47.7)	88523.5 (71127.9, 108330.9)	99.6 (80.2, 121.6)	−8.3 (−13.9, −2.3)
Western Europe	22783.9 (16631.6, 30,904)	1.8 (1.4, 2.4)	30.6 (16.1, 44.3)	5879101.2 (5438217.2, 6289341.4)	737.4 (683, 790.1)	−10.7 (−14.4, −8)	228491.5 (212548.2, 245901.5)	23.9 (22.2, 25.7)	6.2 (−0.6, 13.7)	428631.8 (343313.7, 532188.1)	40.9 (32.9, 49.7)	5.6 (−1.4, 12.7)
Andean Latin America	8378.1 (6459.8, 10828.8)	14.9 (11.5, 19.2)	42.8 (15.1, 76.5)	597138.7 (538460.7, 662178.6)	957.2 (862.9, 1056.2)	−2.7 (−5.8, 0.3)	18007.8 (16150.5, 20130.5)	30.5 (27.4, 34.1)	85.9 (70.3, 103.6)	165500.1 (127469.3, 211085.3)	286.1 (220.7, 365.1)	36.4 (11.6, 68.7)
Central Latin America	24359.4 (19319.3, 30465.4)	10.1 (8, 12.5)	49.9 (37, 64.6)	3407649.4 (3135739.8, 3,667,865)	1327.6 (1,225, 1427.2)	−5.7 (−8.3, −3.3)	106519 (98854.9, 114,268)	41.3 (38.4, 44.3)	45.1 (32.8, 59.5)	582669.3 (462738.3, 718465.8)	232.6 (185.5, 287.1)	56.6 (41.8, 72)
Caribbean	6,205 (5201.2, 7490.7)	11.4 (9.5, 13.7)	43 (24, 61.8)	563202.6 (515384.1, 614522.4)	1069.9 (978.5, 1169.7)	−4.1 (−6.9, −1.4)	14678.6 (13310.5, 16052.2)	27.2 (24.7, 29.8)	62.8 (51.6, 75)	131435.5 (108776.6, 159205.2)	242.9 (200.8, 294.5)	40.1 (21.4, 59)
Tropical Latin America	17882.6 (15002.2, 21195.2)	7.2 (6, 8.6)	16.4 (10.2, 21.5)	2960767.5 (2714465.1, 3207228.6)	1145.8 (1053.3, 1238.7)	−9.5 (−11.9, −7.5)	66,483 (60784.2, 72039)	25.5 (23.3, 27.6)	32.8 (23.1, 45.4)	397813.6 (335704.9, 457742.6)	155.9 (131.5, 178.9)	8.6 (3.6, 13)
High-income North America	57161.5 (49,314, 64516.7)	8.1 (7, 9.1)	259.9 (210.8, 316.3)	6203134.3 (5758563.3, 6652331.6)	1056.5 (979.6, 1135.3)	4.9 (3.5, 6.4)	216863.7 (198070.3, 236861.5)	32.4 (29.7, 35.2)	7.9 (0.6, 16.5)	1153656 (1037947.2, 1260922.9)	174 (157, 190.9)	168.4 (142.9, 199.9)
Central Sub-Saharan Africa	3564.2 (2464.6, 4978.1)	9 (6.1, 12.9)	4.8 (−22.4, 36.5)	976815.1 (874265.7, 1085754.1)	1377.5 (1262.9, 1506.7)	−6.9 (−9.8, −4.1)	6880.6 (5958.1, 7821.2)	12.6 (11.1, 14.2)	36.4 (26.6, 47.3)	97641.6 (69611.5, 134131.5)	196.1 (139.9, 268.7)	2.4 (−20.6, 31.3)
North Africa and Middle East	30956.2 (23,711, 39766.7)	8.2 (6.3, 10.5)	28.9 (−18.2, 60.8)	8039618.4 (7267275.6, 8805017.9)	1505.9 (1369, 1642.9)	−4 (−6.4, −1.7)	199623.4 (182526.9, 218247.3)	42.8 (39.2, 46.5)	63.4 (54.5, 74)	739708.3 (577376.5, 932697.8)	170.2 (133.9, 214.5)	22.3 (−18.8, 49.4)
South Asia	70319.9 (54707.1, 89923.7)	5.3 (4.1, 6.6)	28.5 (−0.7, 55.5)	25462948.6 (23248463.8, 27987293.9)	1547.3 (1418.4, 1687.6)	−9.7 (−12, −7.5)	267934.4 (239653.9, 294287.9)	17.6 (15.8, 19.4)	20.8 (14.7, 27.3)	1,976,809 (1609612.2, 2453203.1)	134.4 (110.5, 166)	22.1 (−0.6, 43.7)
Eastern Sub-Saharan Africa	15078.5 (12109.1, 18892.2)	12 (9.7, 15)	−4.9 (−18.5, 9.5)	2183334.2 (1987627.8, 2407173.7)	942.8 (861.6, 1032.9)	−1.1 (−4.1, 1.1)	18825.3 (16932.8, 20,750)	11.4 (10.3, 12.5)	25 (17.2, 33.7)	344089.2 (277382.9, 431589.1)	230.4 (187.1, 284.8)	−13.5 (−23.9, −1.4)
Western Sub-Saharan Africa	8131.4 (6057.6, 10852.1)	5.5 (4.1, 7.5)	11.4 (−7.2, 29.3)	3216977.7 (2963597.8, 3492631.2)	1276.3 (1183.6, 1373.8)	−3.9 (−6, −2)	34177.2 (30688.3, 37596.6)	17.3 (15.6, 19)	32.8 (25.7, 40.2)	222798.8 (174986.9, 283096.4)	124.3 (97.5, 159.9)	7.6 (−6.5, 22.6)
Southern Sub-Saharan Africa	2,069 (1552.7, 2770.1)	4.4 (3.3, 5.8)	49.3 (10.3, 73.8)	890370.9 (815493.6, 968328.5)	1362.7 (1254.9, 1,471)	−3.9 (−6.4, −1.6)	13773.6 (12470.5, 15069.9)	23.4 (21.1, 25.5)	40.9 (33.8, 48.8)	60270.1 (47470.2, 78172.5)	108.9 (85.5, 140)	36.9 (11.3, 55)

Gender-specific analysis revealed that in 2021, the number of incidence, prevalence, deaths, and DALYs counts, were higher in males in younger adults, but higher in females in older adults. As for their respective ASRs, it was still higher in males (Supplementary Figures S1–S4).

An age-stratified analysis of incidence, prevalence, deaths, and DALYs in 2021 is presented in Supplementary Figures S1–S4. For the number of cases, there was an initial increase with age, peaking in around older years old, followed by a decrease. For ASIR and ASPR, the disease burden also showed the “N” trend, but the ASDR and ASDAR still increase with age (Supplementary Figures S1–S4).

At the SDI region level, the middle SDI region had the highest number of incidence cases, prevalence cases, deaths cases, DALYs cases, ASIR, ASDR, and ASDAR. For the ASPR, the disease burden was highest in the Low-middle SDI regions (Table 1).

Across the 21 GBD regions, Andean Latin America ranked the top one in ASIR at 14.9 (95% UI: 11.5, 19.2), Southeast Asia ranked the top one in ASPR at 1739.3 (95% UI: 1595.7, 1883.9), East Asia ranked the top one in ASDR at 373610.9 (95% UI: 340685.9, 402345.5), and Oceania ranked the top one in ASDAR at 309.8 (95% UI: 257.3, 383.9). For the number of cases, the top GBD region was South Asia for incidence at 70319.9 (95% UI: 54707.1, 89923.7) and DALYs at 1,976,809 (95% UI: 1609612.2, 2453203.1). The top GBD region for number of prevalence cases was North Africa and Middle East at 8039618.4 (95% UI: 7267275.6, 8805017.9). And the top GBD region for number of deaths cases was East Asia at 373610.9 (95% UI: 340685.9, 402345.5) (Table 1).

**Figure 1:**
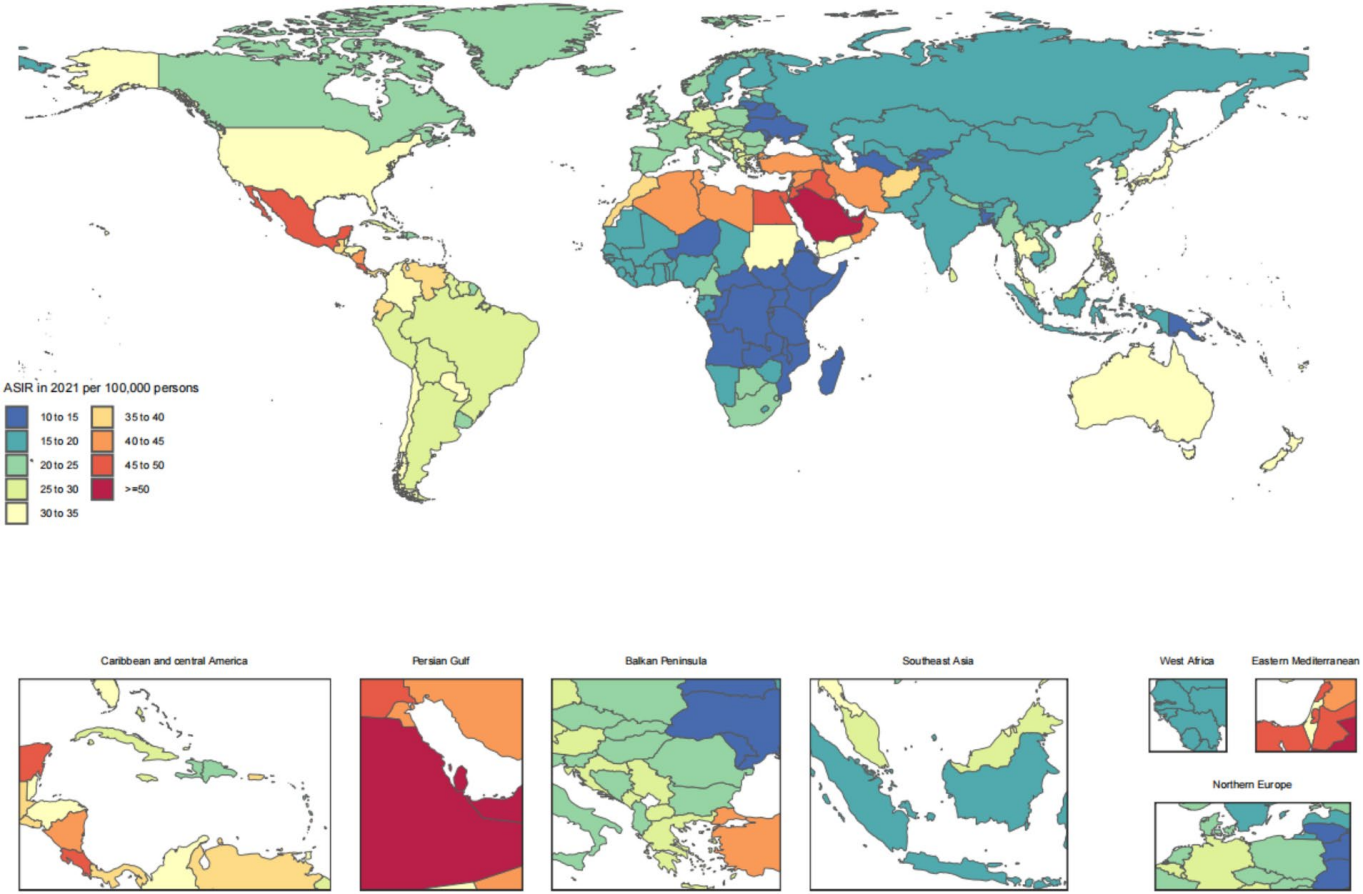
Age-standardized rates of chronic kidney disease due to diabetes mellitus type 2-related incidence across countries and territories in 2021.

**Figure 2:**
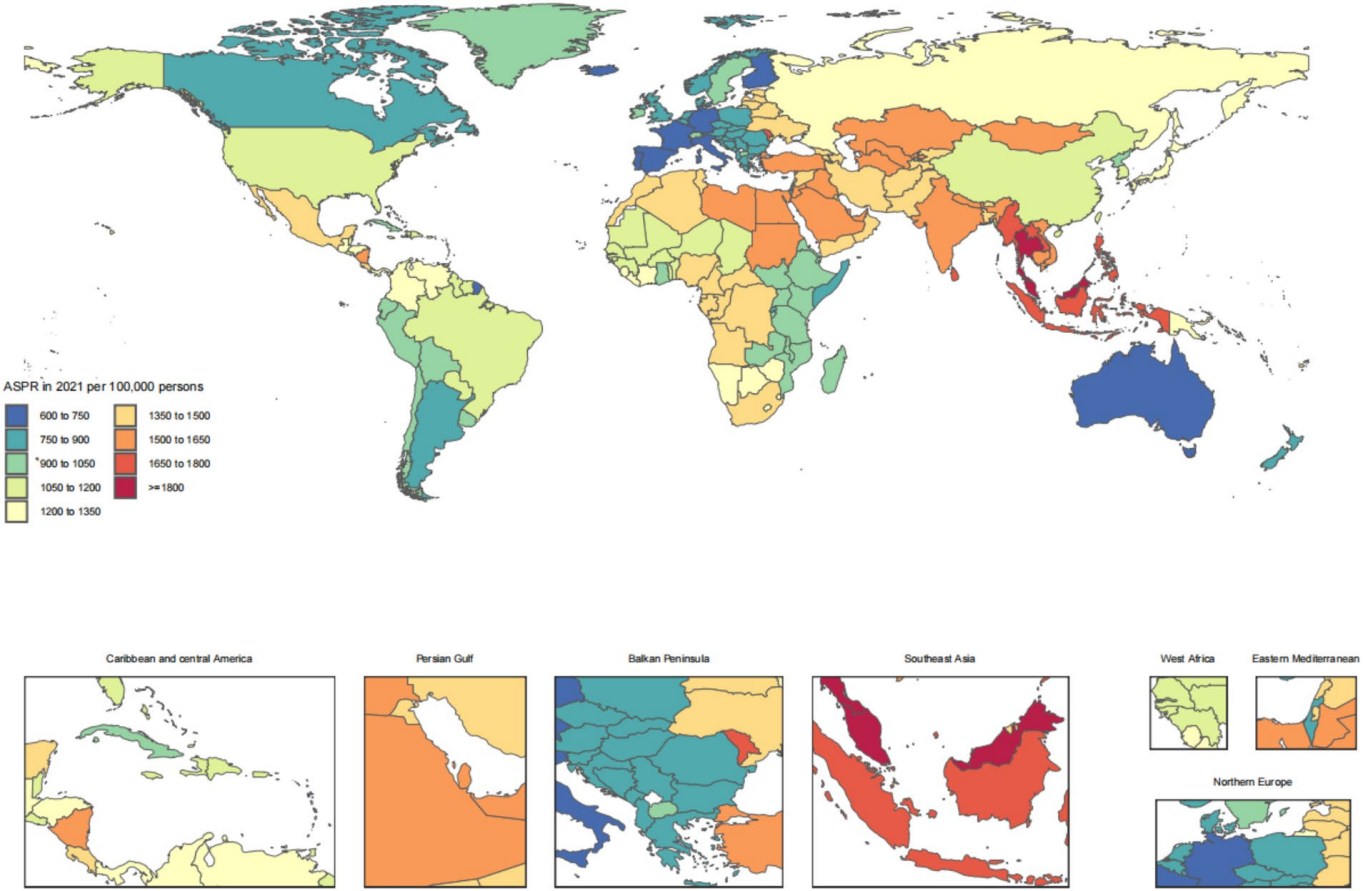
Age-standardized rates of chronic kidney disease due to diabetes mellitus type 2-related prevalence across countries and territories in 2021.

**Figure 3:**
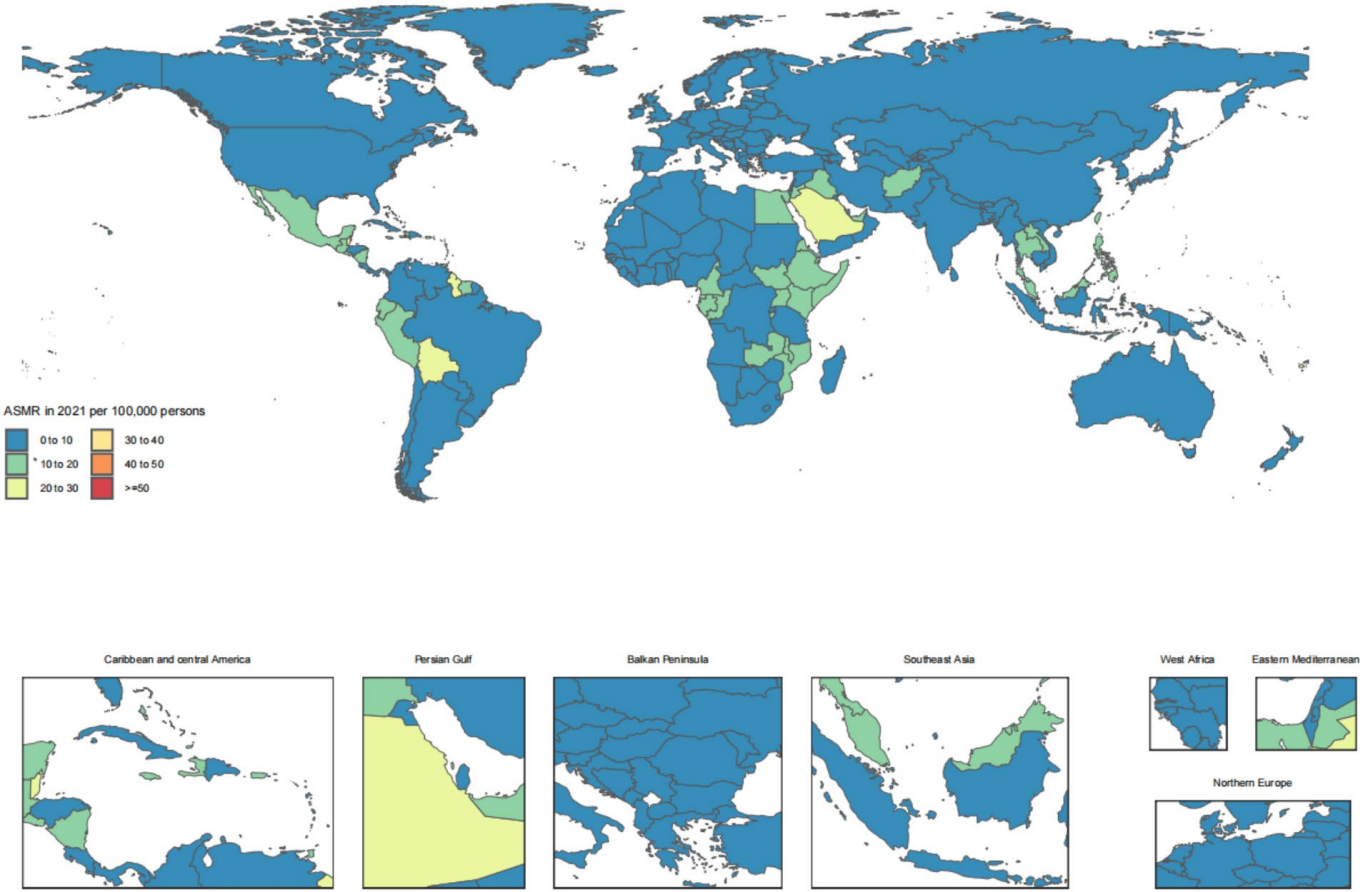
Age-standardized rates of chronic kidney disease due to diabetes mellitus type 2-related deaths across countries and territories in 2021.

**Figure 4:**
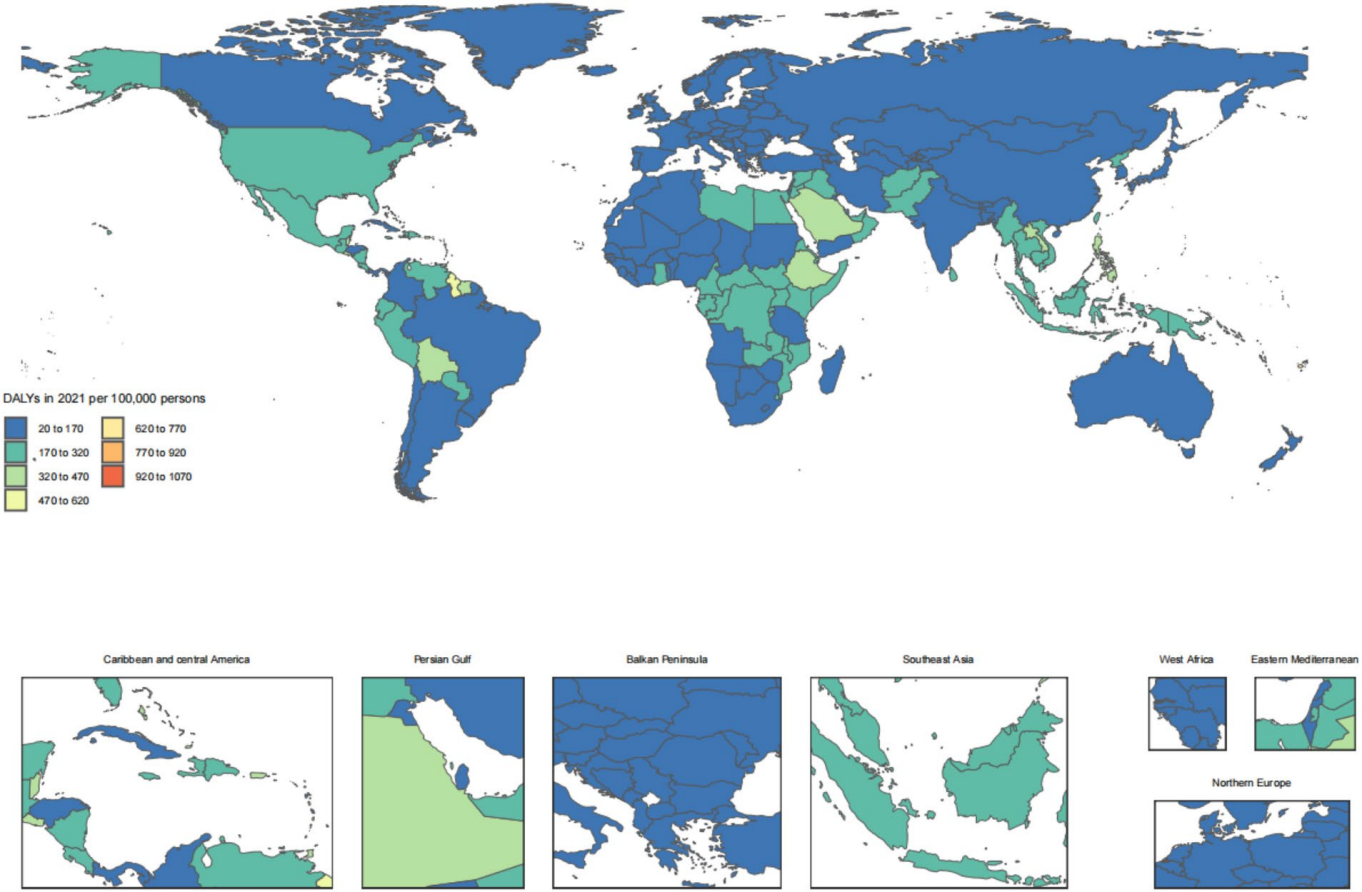
Age-standardized rates of chronic kidney disease due to diabetes mellitus type 2-related DALYs across countries and territories in 2021. DALYs, disability-adjusted life years.

The disease burden of CKD osteoarthritis knee varied considerably across the world, as detailed in Figures 1–4.

### 3.2 Temporal trend for CKD due to T2DM-related disease burden from 1990 to 2021

Globally, the ASRs of CKD due to T2DM all have witnessed a notable rise except for the ASPR. The percentage change in ASRs was 37.8 (95% confidence interval (CI): 19.2, 49.6) for ASIR, was 21 (95% CI: 15, 27.5) for ASDR, and was 24 (95% CI: 9.3, 33) for ASDAR from 1990 to 2021. However, for ASPR, the percentage change was −5.1 (95% CI: −7.5, −3), showed a decreasing trend (Table 1).

In our analysis at the regional level of the SDI, a discernible pattern emerges in the temporal trends of CKD due to T2DM indicators. Regarding the ASRs of incidence and deaths, all SDI regions consistently demonstrate an upward trend. For the ASPR, all SDI regions showed the decreasing trend. However, for ASDAR, all SDI regions showed the increasing trend except for Low SDI regions (Table 1). From 1990 to 2021, the deaths and DALYs of CKD due to T2DM both for males and females in high SDI region were lowest in 1990 compared to other SDI regions, which increased significantly to the extent that, around 2010, exceeded those in the high-middle SDI regions. Notably, the deaths and DALYs of CKD due to T2DM both for males and females presented a downward trend in low SDI region during 2000 to 2010. Detailed information is depicted in Supplementary Figure S13. Between 1990 and 2021, the ASIR of CKD due to T2DM increased steadily among both men and women across all SDI regions. In contrast, the ASPR of CKD due to T2DM decreased consistently for both genders in all SDI regions during the same period. See Supplementary Figure S14 for details.

We further elucidate the variability in CKD due to T2DM burden across GBD regions, the results were presented in Table 1. The most pronounced increase in ASIR, ASPR, and ASDAR from 1990 to 2021 was observed in High-income North America (ASIR: percentage change = 259.9, 95% CI: 210.8, 316.3; ASPR: percentage change = 4.9, 95% CI: 3.5, 6.4; ASDAR: percentage change = 168.4, 95% CI: 142.9, 199.9), and the most pronounced increase was observed in Andean Latin America for ASDR (percentage change = 85.9, 95% CI: 70.3, 103.6) (Table 1).

### 3.3 The regular pattern of different SDI levels and CKD due to T2DM-related disease burden

**Figure 5:**
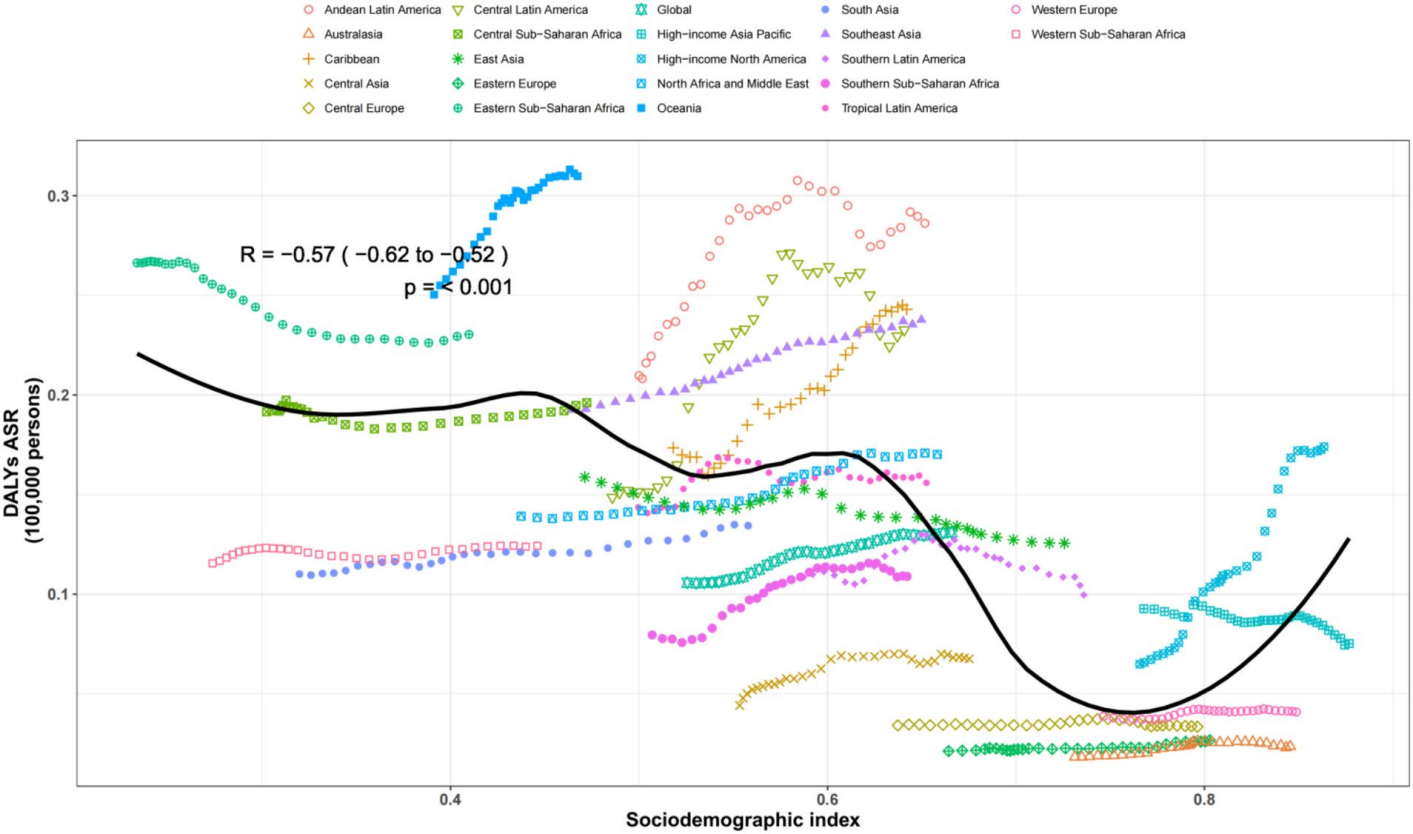
Age-standardized rates of DALYs attributable to chronic kidney disease due to diabetes mellitus type 2 across regions by socio-demographic index for both sexes, 1990–2019. The black line was an adaptive association fitted with adaptive LOESS regression based on all data points. DALYs, disability-adjusted-life-years.

**Figure 6:**
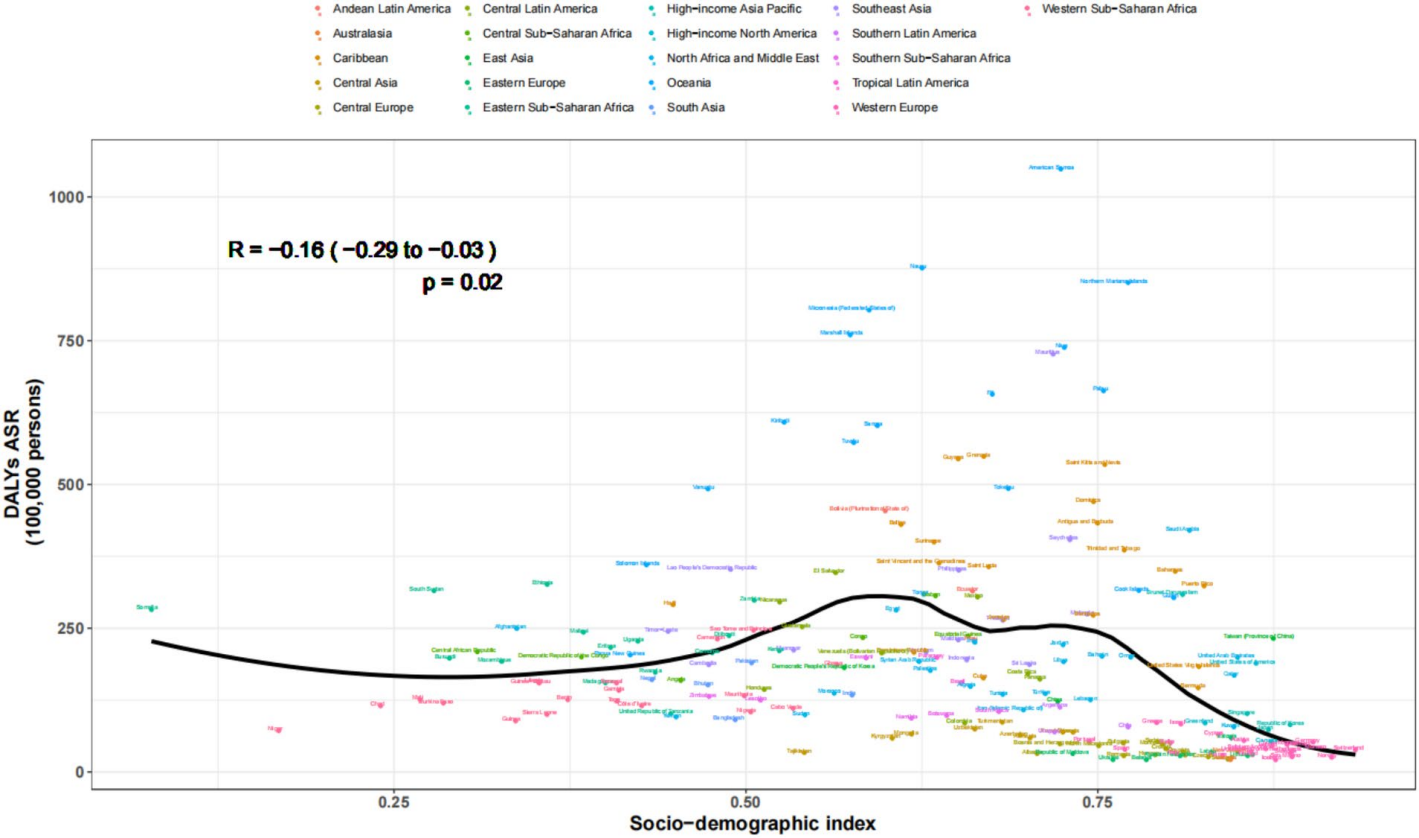
Age-standardized rates of DALYs attributable to chronic kidney disease due to diabetes mellitus type 2 across countries and territories by socio-demographic index for both sexes, 1990-2021.

The regular pattern of different SDI levels and the ASDAR was stable across countries and territories. From Figures 5, 6, the ASRs for DALYs showed a negative correlation with SDI, indicating that higher SDI indicates lower disease burden (Figures 5, 6).

### 3.4 The predicted results from 2022 to 2036

The projections by the BAPC model indicate an upward trajectory in the ASRs for over the period spanning from 2022 to 2036. Notably, the ASIR, ASDR, and ASDAR for both genders were increasing, however, for the ASDAR, it showed a decreasing trend (Supplementary Figures S5–S12).

## 4 Discussion

As far as we know, this was the latest study to comprehensively assess and quantify CKD due to T2DM-related disease burden globally. From 1990 to 2021, there was an increasing trend for the disease burden globally and for sub-types including sexes and across ages, SDI regions, GBD regions, and countries. Furthermore, our predicted results showed that the ASRs would still increase in the next 15 years.

In 2021, the disease burden of CKD due to T2DM emerged as a significant global health challenge. Our findings align with previous research indicating a rising trend in T2DM-related CKD incidence, prevalence, and mortality (8, 30). Notably, the age-standardized incidence and prevalence rates reported here underscore the substantial and growing impact of this comorbidity, surpassing estimates from earlier studies that highlighted a growing burden but at lower magnitudes (12, 31). The mortality rate and DALYs associated with T2DM-CKD further emphasize the dire need for effective interventions and management strategies. Comparisons with data from the Global Burden of Disease Study demonstrate consistency in the direction of these trends but suggest potential regional variations that warrant detailed investigation (32). Addressing modifiable risk factors such as glycemic control, blood pressure management, and lifestyle modifications could significantly mitigate this burden, as echoed in contemporary literature (33, 34). Continued surveillance and targeted public health initiatives are crucial to curb the escalating disease burden of T2DM-CKD globally.

Our findings reveal a compelling temporal trend in the disease burden of CKD due to T2DM globally from 1990 to 2021. The notable rise in ASIR, ASDR, and ASDAR aligns with previous studies indicating an increasing prevalence and severity of T2DM-related complications (35, 36). However, the decrease in ASPR observed in our study contrasts with some earlier reports, which may be attributed to improvements in early detection, management, and survival rates among CKD patients (12, 31). This trend suggests a complex interplay between disease incidence, progression, and mortality. The rising ASIR and ASDR underscore the urgent need for effective interventions to mitigate the growing burden of T2DM-CKD, while the decreasing ASPR may reflect advancements in healthcare delivery and patient care. Future research should focus on understanding these trends further and identifying strategies to reduce the overall disease burden globally.

The gender-specific analysis unveils intriguing patterns in the disease burden of the studied condition across different age groups. The finding that in 2021, incidence, prevalence, deaths, and DALYs were higher in males among younger adults aligns with prior research suggesting that males in this demographic may face greater exposure to risk factors such as unhealthy lifestyles and occupational hazards (37). Conversely, the shift towards higher burden in females among older adults could be attributed to biological factors, including hormonal changes and differences in immune responses, as well as socio-cultural practices that may affect healthcare access and utilization (38, 39). Despite these age-specific gender disparities, the consistently higher ASRs in males across various metrics emphasize the need for tailored interventions that account for gender-specific vulnerabilities and risk profiles. Further research is required to elucidate the underlying mechanisms driving these gender-and age-related differences and to develop comprehensive strategies to reduce the overall disease burden in both males and females.

The age-stratified analysis of incidence, prevalence, deaths, and DALYs in our study reveals intriguing patterns that align with and extend the findings of previous research (40, 41). The initial rise in case numbers with age, followed by a decline in older age groups, may reflect complex interplay between exposure risk, immune response, and survival factors. This trend is consistent with observations in other chronic conditions, where elderly populations often exhibit higher incidence but may be under-represented in case counts due to higher mortality rates (42). Notably, the ASIR and ASPR exhibit an “N”-shaped trend, suggesting a peak incidence and prevalence in middle to older age groups, followed by a decline. This pattern contrasts with the continuous increase in ASDR and ASDAR with advancing age, highlighting the increasing severity and burden of disease in older populations (43). These findings underscore the importance of age-specific interventions and highlight the need for tailored healthcare strategies that address the unique needs of different age groups. Our results contribute to the growing body of evidence emphasizing the critical role of age in determining disease burden and outcomes. Future research should continue to explore the underlying biological and sociodemographic factors driving these age-related trends to inform more effective public health policies and interventions (44, 45).

The findings reveal notable disparities in the burden of CKD due to T2DM across different SDI regions. The middle SDI regions exhibited the highest burden in terms of incidence, prevalence (except for low-middle SDI regions which had the highest ASPR), deaths, DALYs, ASIR, ASDR, and ASDAR. These observations align with previous studies indicating that middle-income countries often face a double burden of communicable and non-communicable diseases, including diabetes-related complications (46, 47). The upward trend in incidence and death rates across all SDI regions underscores the global surge in diabetes-related CKD, possibly due to urbanization, sedentary lifestyles, and unhealthy diets (4). The decreasing ASPR in all regions, except for the increasing ASDAR in middle and high SDI regions (excluding Low SDI), suggests improvements in case management and survival but highlights persistent morbidity and mortality risks, particularly among older and more vulnerable populations (12, 48). These findings emphasize the need for tailored interventions targeting high-risk populations and strengthening healthcare systems to manage the growing burden of diabetes-related CKD effectively.

The findings of our study reveal striking regional disparities in the burden of CKD due to T2DM across the 21 GBD regions. Notably, Andean Latin America exhibited the highest ASIR, aligning with previous observations that highlighted the rising prevalence of diabetes and its complications in this region (49). Southeast Asia topped the list for ASPR, potentially linked to high-risk behaviors and limited access to healthcare services, as reported in existing literature (50). East Asia displayed the highest ASDR and number of deaths, reflecting the aging population and the increasing incidence of diabetes-related complications (51). Oceania showed the highest ASDAR, suggesting a substantial impact on health outcomes and quality of life (12). South Asia led in terms of incidence cases and DALYs, possibly due to a combination of high population density, lifestyle changes, and inadequate healthcare infrastructure (52). North Africa and the Middle East reported the highest prevalence cases, consistent with studies highlighting the regional burden of CKD and its association with diabetes (31). Furthermore, our analysis revealed High-income North America experienced the most significant increase in ASIR, ASPR, and ASDAR from 1990 to 2021, indicating a growing public health challenge despite advanced healthcare systems (53). Andean Latin America showed the largest increase in ASDR, emphasizing the urgent need for interventions to mitigate the diabetes epidemic in this region (49). These findings underscore the necessity for tailored public health strategies to address the regional variations in the CKD burden due to T2DM.

The observed regular pattern of different SDI levels and the ASDAR associated with CKD due to T2DM reveals a consistent trend across various countries and territories. Our findings align with previous studies suggesting that higher SDI levels are inversely related to the disease burden, indicating better healthcare access and management in higher-SDI countries (54, 55). This negative correlation between SDI and the ASDAR underscores the role of socioeconomic factors in influencing disease outcomes. Furthermore, the stability of this pattern across diverse regions suggests that global health policies aimed at mitigating the T2DM-related kidney disease burden should consider socioeconomic disparities (4, 31). Future interventions should prioritize resource allocation and healthcare infrastructure development in lower-SDI settings to reduce health inequalities.

The projected upward trajectory in ASIR, ASDR, and overall disease burden, as indicated by our BAPC model for the period 2022 to 2036, aligns with existing literature suggesting a growing global health burden related to chronic conditions (8, 14). This increasing trend in ASIR and ASDR is particularly concerning, as it highlights the need for intensified preventive measures and improved management strategies. However, crucially, the early detection and management of diabetes, hypertension, and chronic kidney disease (CKD) can be achieved through the use of widely accessible and often low-cost interventions. These measures have the potential to enhance renal and cardiovascular outcomes, as well as to delay or prevent the progression to end-stage kidney disease (ESKD) (12). The decrease observed in ASDAR suggests potential improvements in disease management and quality of life for those affected, healthcare system gradually paid more attention to early stages of CKD rather than the treatment for ESKD, which indicating advancements in healthcare and treatment options (12), eventually resulted in the decrease of ASDAR. Furthermore, given the substantial burden of CKD in middle-and low-income countries, on the one hand, building a tiered healthcare system is key to addressing the challenges posed by CKD. By strengthening primary healthcare services, CKD can be detected early and intervened in a timely manner, thereby reducing the reliance of severe patients on high-end medical resources in the later stages. On the other hand, Policy-making bodies can alleviate the disease burden of CKD by strengthening public health education and awareness (56), including raising public awareness of the early symptoms of CKD, encouraging regular screenings and early diagnoses, and promoting the expansion of social security coverage, which is especially important in low SDI regions where primary care health systems are less equipped to adequately prevent and treat chronic diseases (57). These mixed trends underscore the complexity of the disease burden and the importance of multifaceted approaches to address both incidence and mortality while enhancing patient outcomes. Future research should focus on identifying the drivers of these trends and developing targeted interventions to mitigate the projected increase in disease burden.

As we know, in clinical practice, a multimodal intervention strategy using all available tools to target a major pathogenic factor in the progression of CKD such as proteinuria seems a rational approach to maximizing renoprotection in CKD patients, including lifestyle modifications such as sodium and protein intake restriction, smoking cessation, body weight loss, optimal BP (target systolic/diastolic, 130/80 mmHg) and metabolic control (target hemoglobin A1C, 7.5%) in patients with diabetes, correction of metabolic acidosis and hyperphosphatemia, use of statins and dual renin-angiotensin system (RAS) blockade with maximum tolerated doses of angiotensin converting enzyme (ACE) inhibitors and angiotensin II receptor blockers (ARBs), probably the mainstay of proteinuria management in this setting (58). A balanced diet and healthy lifestyle habits can directly prevent or alleviate the symptoms of CKD. Additionally, they can indirectly reduce the disease burden of CKD through their mediating effect on lowering the incidence of T2DM.

While our study features a comprehensive framework for data collection and analysis, it is crucial to acknowledge several inherent limitations. Firstly, Variations in the availability and quality of data across different countries and regions may introduce biases into our estimates (59). GBD relies on statistical methods and predictive covariate values to estimate the CKD burden in regions with unavailable data on CKD incidence or prevalence. Sources of non-fatal CKD data vary in terms of sampling, laboratory techniques, and the equations used to calculate estimated glomerular filtration rate (eGFR), leading to systematic discrepancies in CKD prevalence estimates due to the use of different equations (60, 61). Additionally, the evolution of diagnostic criteria and advancements in treatment technologies over time could potentially compromise the accuracy of historical comparisons (62). Lastly, it is inherent to projections that they entail a degree of uncertainty, and therefore, they should be interpreted with caution (63).

## 5 Conclusion

In conclusion, CKD due to T2DM poses a considerable global health burden, particularly in regions characterized by middle and lower economic development, underscoring its wide-ranging implications. Furthermore, our projections indicate a persistent upward trend in the ASRs from 2022 to 2036 except for the prevalence, emphasizing the continued significance of CKD due to T2DM as a pressing public health challenge that necessitates urgent attention.

## Data Availability

The original contributions presented in the study are included in the article/Supplementary material, further inquiries can be directed to the corresponding authors.

## Footnotes

^1^ https://vizhub.healthdata.org/gbd-results/
