# Supplementary material for: Global, regional, and national burden of chronic kidney disease due to diabetes mellitus type 2 from 1990 to 2021, with projections to 2036: a systematic analysis for the Global Burden of Disease Study 2021
Source: Front Med (Lausanne). 2025 Feb 17;12:1531811. doi: 10.3389/fmed.2025.1531811 (PMC11872908; doi:10.3389/fmed.2025.1531811)
Supplement: Supplementary file 1 [file Data_Sheet_1.pdf]

Global, regional, and national burden of chronic kidney disease due to diabetes mellitus type 2 from 1990 to 2021, with projections to 2036: a systematic analysis for the Global Burden of Disease Study 2021

Figure legend

Figure S1. Age-standardized rates of chronic kidney disease due to diabetes mellitus type 2-related incidence for different age groups by both sexes in 2021.

Figure S2. Age-standardized rates of chronic kidney disease due to diabetes mellitus type 2-related prevalence for different age groups by both sexes in 2021.

Figure S3. Age-standardized rates of chronic kidney disease due to diabetes mellitus type 2-related deaths for different age groups by both sexes in 2021.

Figure S4. Age-standardized rates of chronic kidney disease due to diabetes mellitus type 2-related DALYs for different age groups by both sexes in 2021. Abbreviations: DALYs, disability-adjusted life years.

Figure S5. The predicted results in the chronic kidney disease due to diabetes mellitus type 2-related age-standardized rates of incidence for male globally from 2022 to 2036 of the BAPC model. Abbreviations: BAPC, Bayesian age-period-cohort.

Figure S6. The predicted results in the chronic kidney disease due to diabetes mellitus type 2-related age-standardized rates of prevalence for male globally from 2022 to 2036 of the BAPC model. Abbreviations: BAPC, Bayesian age-period-cohort.

Figure S7. The predicted results in the chronic kidney disease due to diabetes mellitus type 2-related age-standardized rates of deaths for male globally from 2022 to 2036 of the BAPC model. Abbreviations: BAPC, Bayesian age-period-cohort.

Figure S8. The predicted results in the chronic kidney disease due to diabetes mellitus type 2-related age-standardized rates of DALYs for male globally from 2022 to 2036 of the BAPC model. Abbreviations: DALYs, disability-adjusted-life-year; BAPC, Bayesian age-period-cohort.

Figure S9. The predicted results in the chronic kidney disease due to diabetes mellitus type 2-related age-standardized rates of incidence for female globally from 2022 to 2036 of the BAPC model. Abbreviations: BAPC, Bayesian age-period-cohort.

Figure S10. The predicted results in the chronic kidney disease due to diabetes mellitus type 2-related age-standardized rates of prevalence for female globally from 2022 to 2036 of the BAPC model. Abbreviations: BAPC, Bayesian age-period-cohort.

Figure S11. The predicted results in the chronic kidney disease due to diabetes mellitus type 2-related age-standardized rates of deaths for female globally from 2022 to 2036 of the BAPC model. Abbreviations: BAPC, Bayesian age-period-cohort.

Figure S12. The predicted results in the chronic kidney disease due to diabetes mellitus type 2-related age-standardized rates of DALYs for female globally from 2022 to 2036 of the BAPC model. Abbreviations: DALYs, disability-adjusted-life-year; BAPC, Bayesian age-period-cohort.

Figure S13. Trends in mortality and DALYs of chronic kidney disease due to diabete s mellitus type 2 stratified by SDI regions and gender from 1990 to 2021.

Figure S14. Trends in ASIR and ASPR of chronic kidney disease due to diabetes mel litus type 2 stratified by SDI regions and gender from 1990 to 2021.

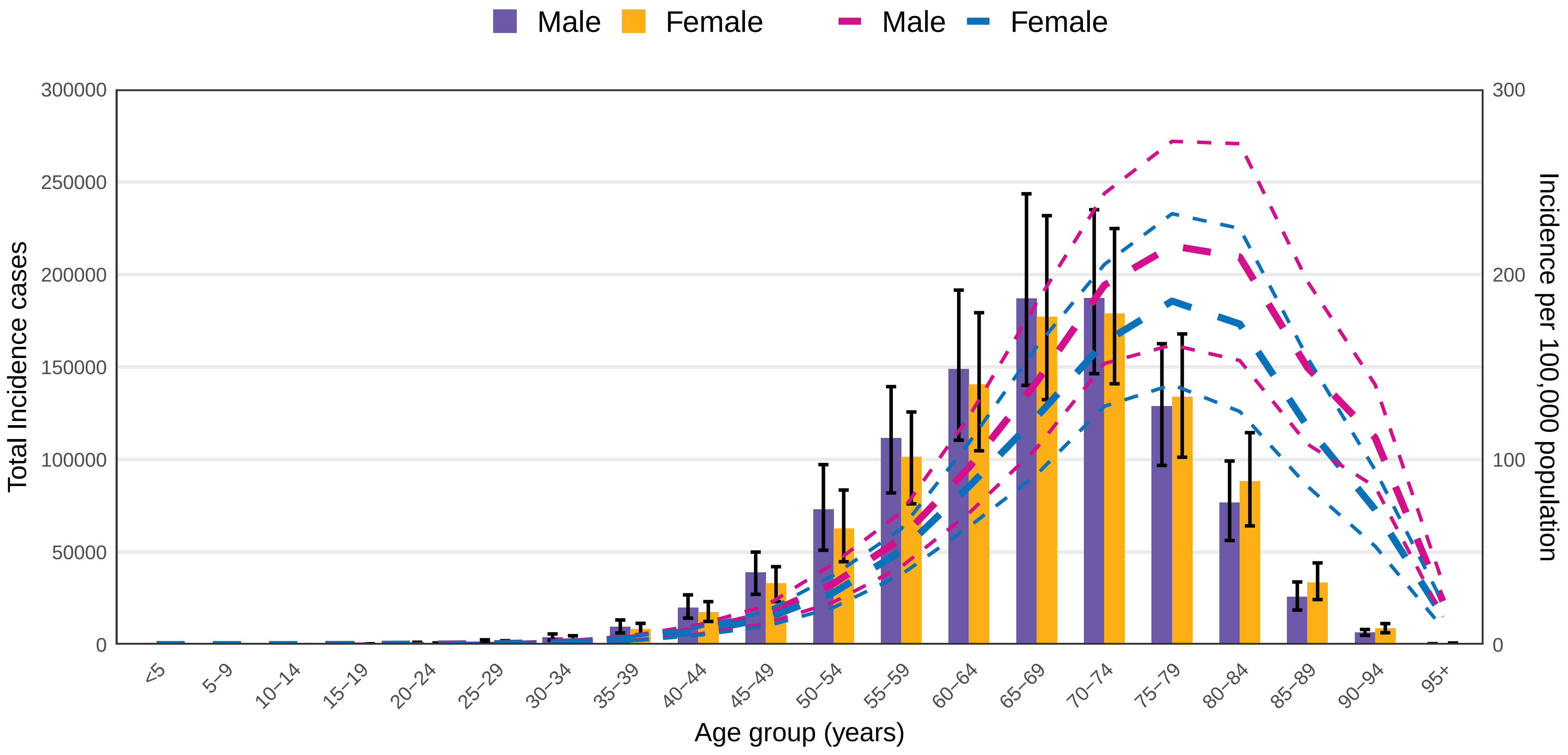

Male
Female
Male
Female
Total Incidence cases
300000
250000
200000
150000
100000
50000
0
300
200
100
0
Incidence per 100,000 population
<5
5–9
10–14
15–19
20–24
25–29
30–34
35–39
40–44
45–49
50–54
55–59
60–64
65–69
70–74
75–79
80–84
85–89
90–94
95+
Age group (years)

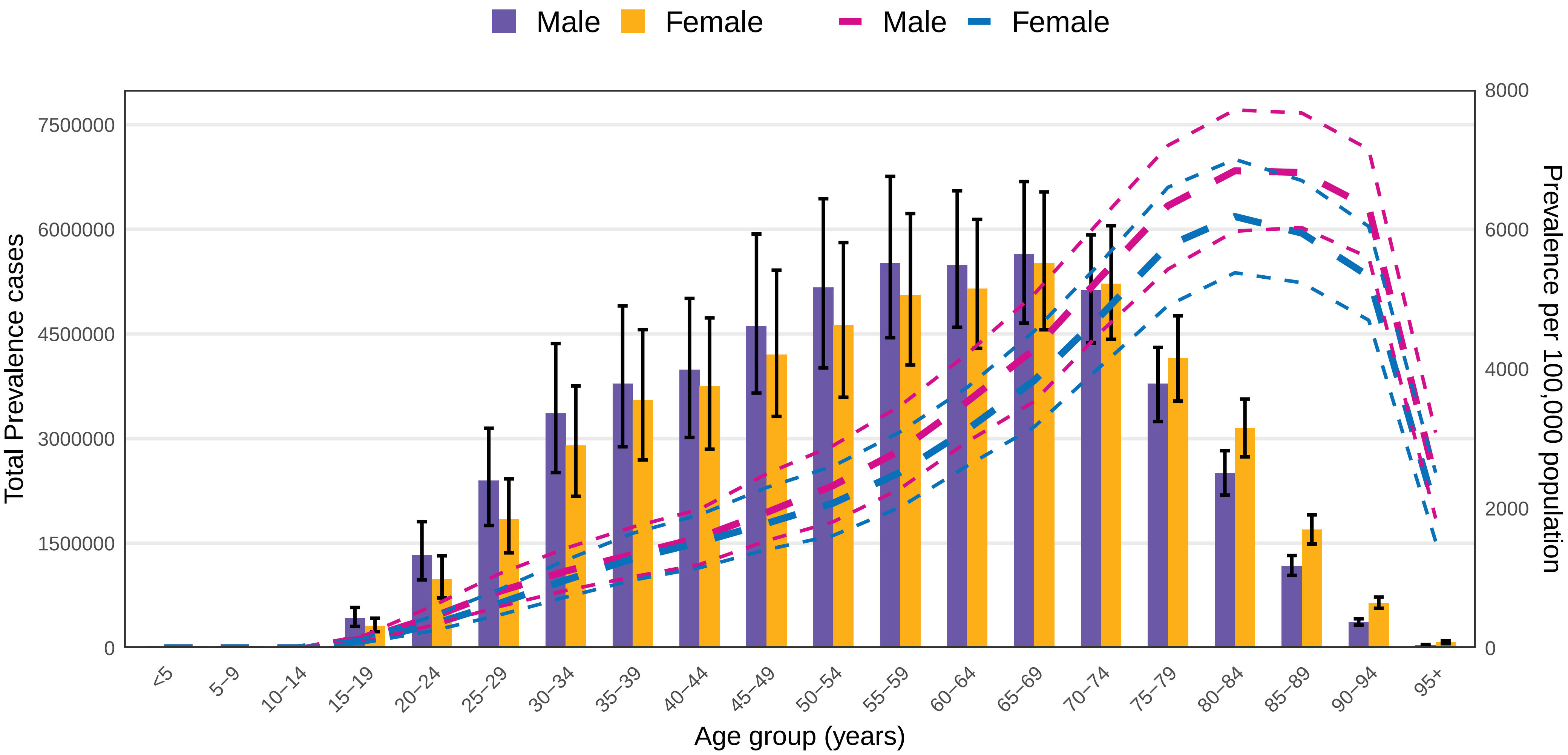

Male
Female
Male
Female
Total Prevalence cases
Prevalence per 100,000 population
Age group (years)
7500000
6000000
4500000
3000000
1500000
0
8000
6000
4000
2000
0
<5
5–9
10–14
15–19
20–24
25–29
30–34
35–39
40–44
45–49
50–54
55–59
60–64
65–69
70–74
75–79
80–84
85–89
90–94
95+

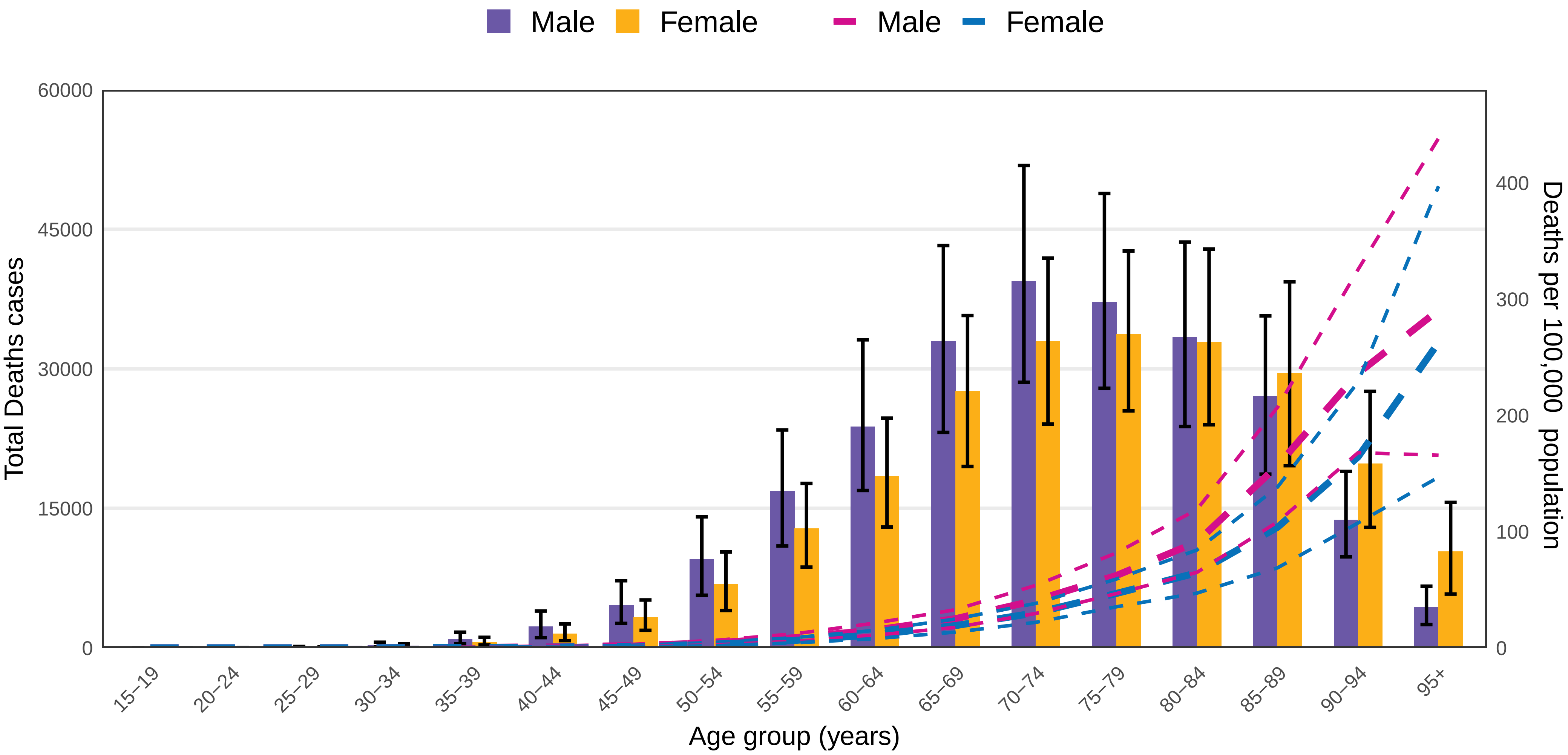

Male
Female
Male
Female
Total Deaths cases
Deaths per 100,000 population
60000
45000
30000
15000
0
400
300
200
100
0
15–19
20–24
25–29
30–34
35–39
40–44
45–49
50–54
55–59
60–64
65–69
70–74
75–79
80–84
85–89
90–94
95+
Age group (years)

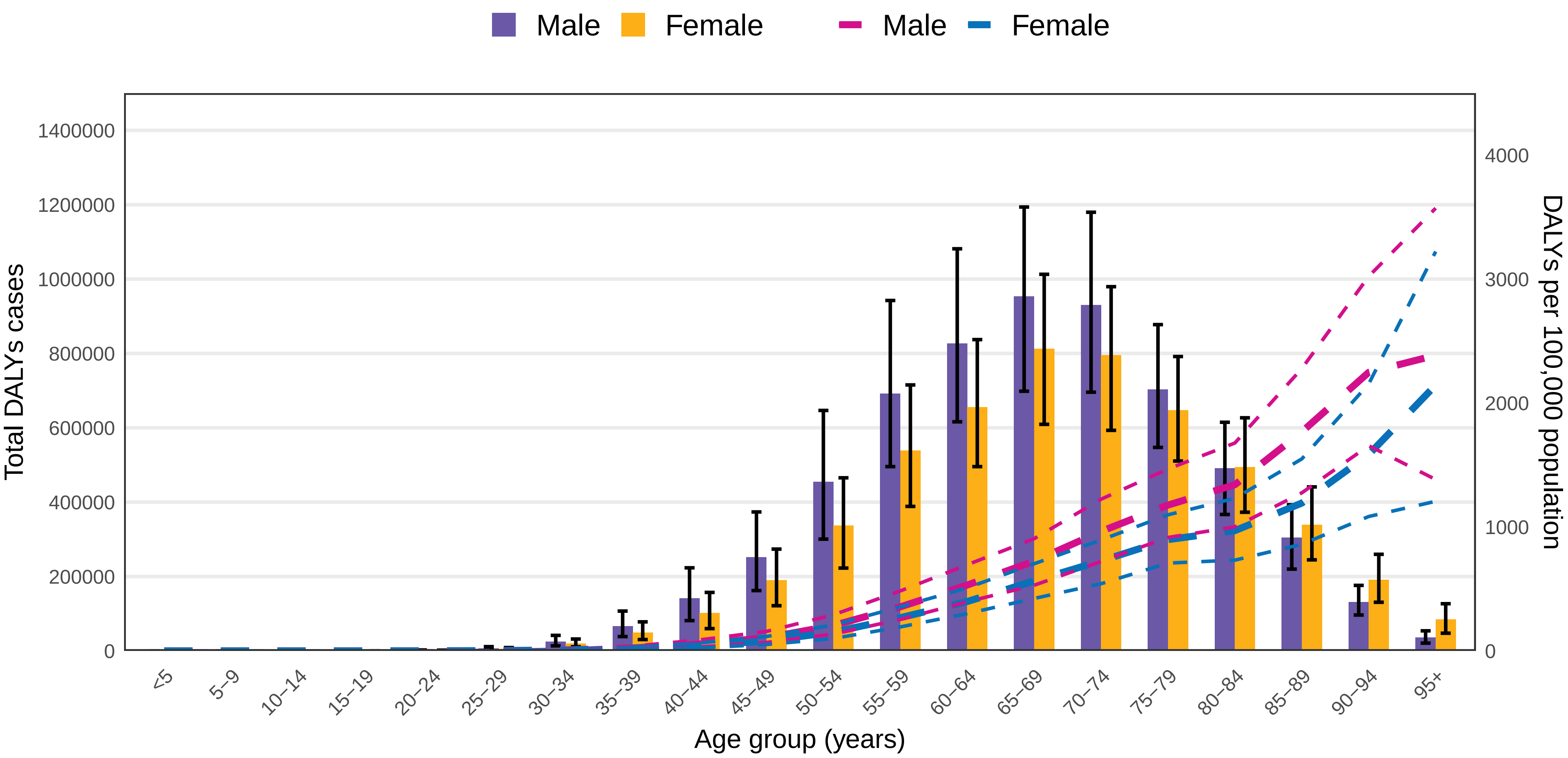

Male
Female
Male
Female
Total DALYs cases
0
200000
400000
600000
800000
1000000
1200000
1400000
DALYs per 100,000 population
0
1000
2000
3000
4000
<5
5–9
10–14
15–19
20–24
25–29
30–34
35–39
40–44
45–49
50–54
55–59
60–64
65–69
70–74
75–79
80–84
85–89
90–94
95+
Age group (years)

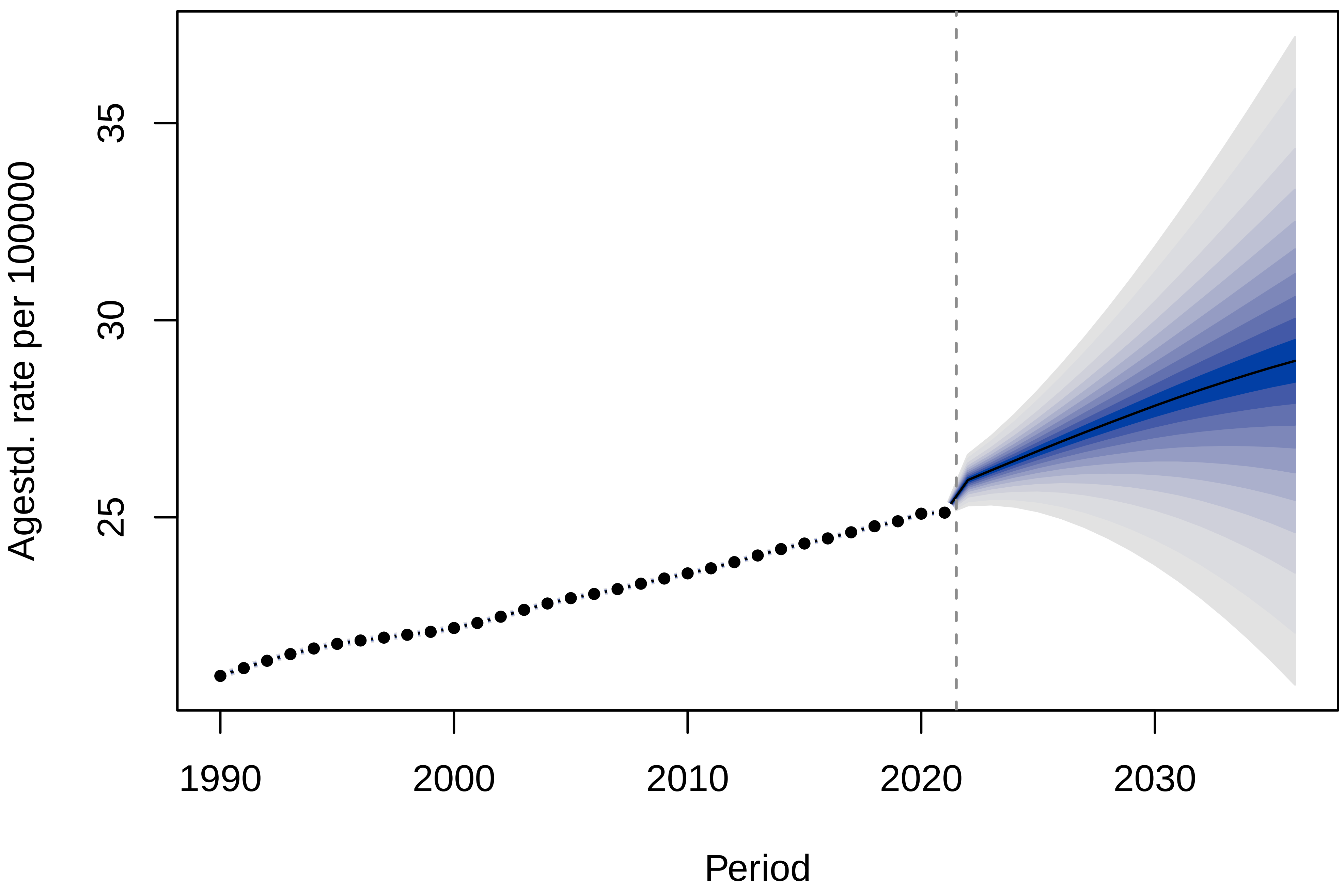

Agestd. rate per 1000000
25
30
35
1990
2000
2010
2020
2030
Period

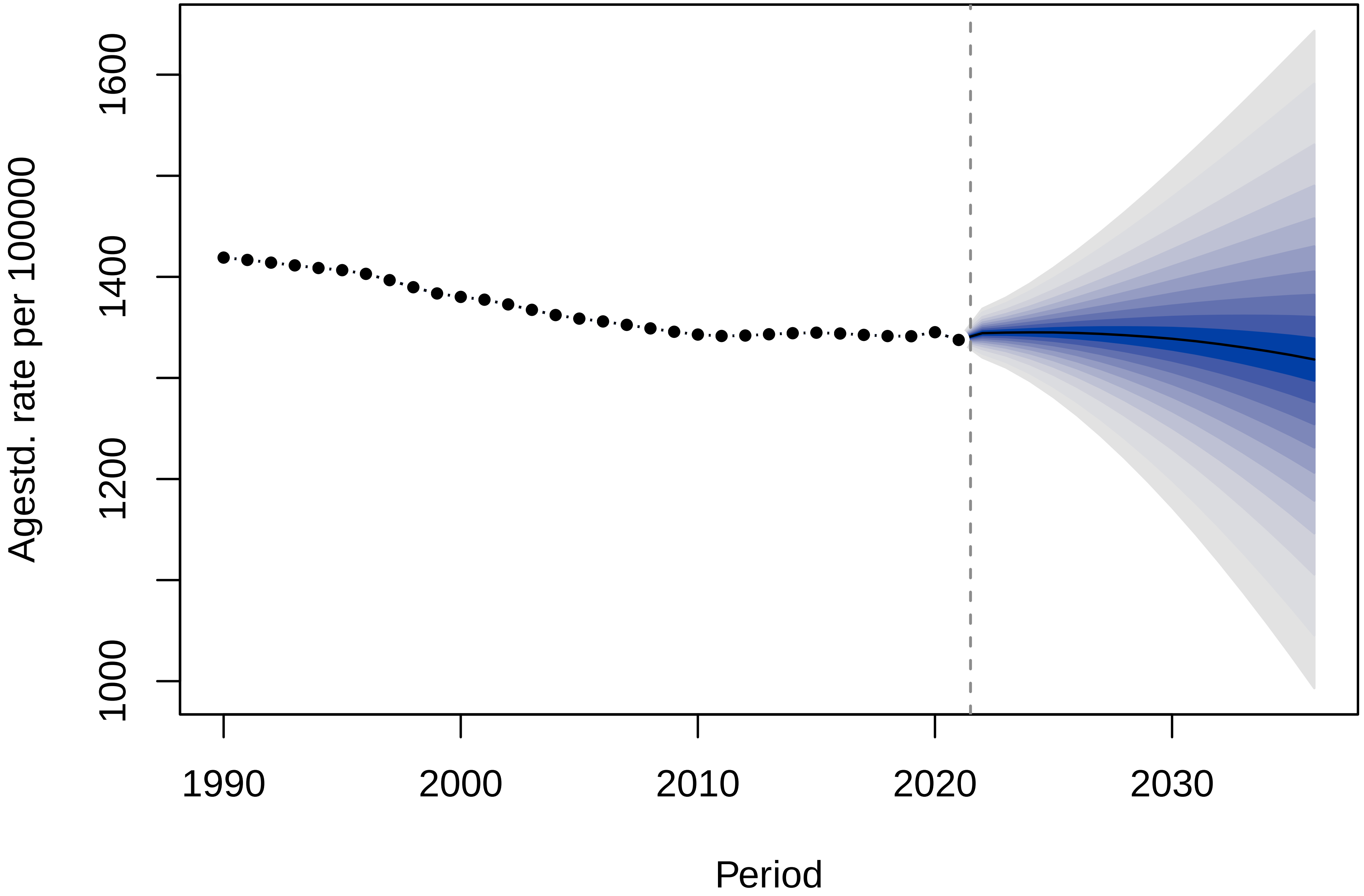

Agestd. rate per 1000000
1000
1200
1400
1600
1990
2000
2010
2020
2030
Period

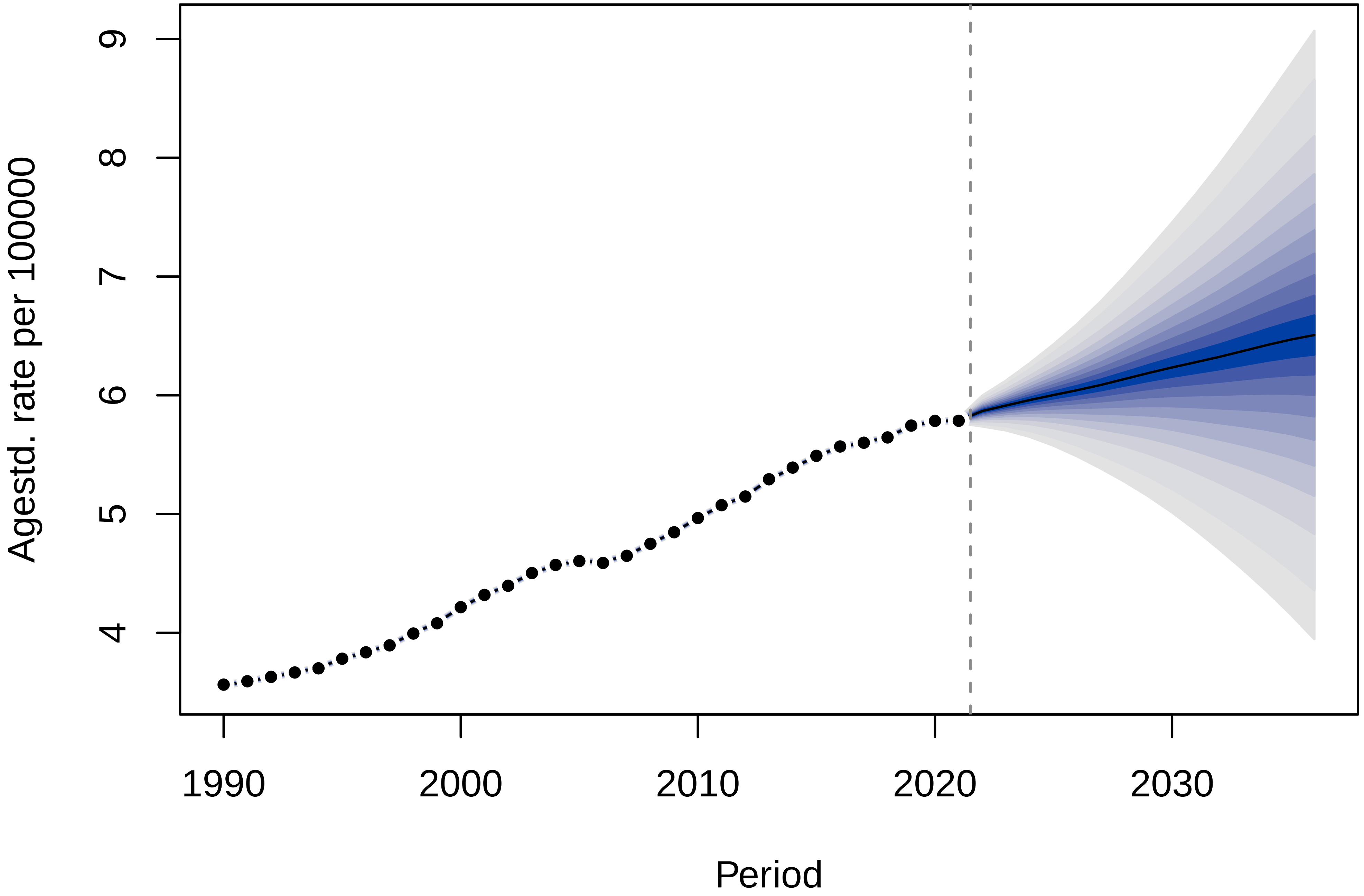

Agestd. rate per 1000000
9
8
7
6
5
4
1990
2000
2010
2020
2030
Period

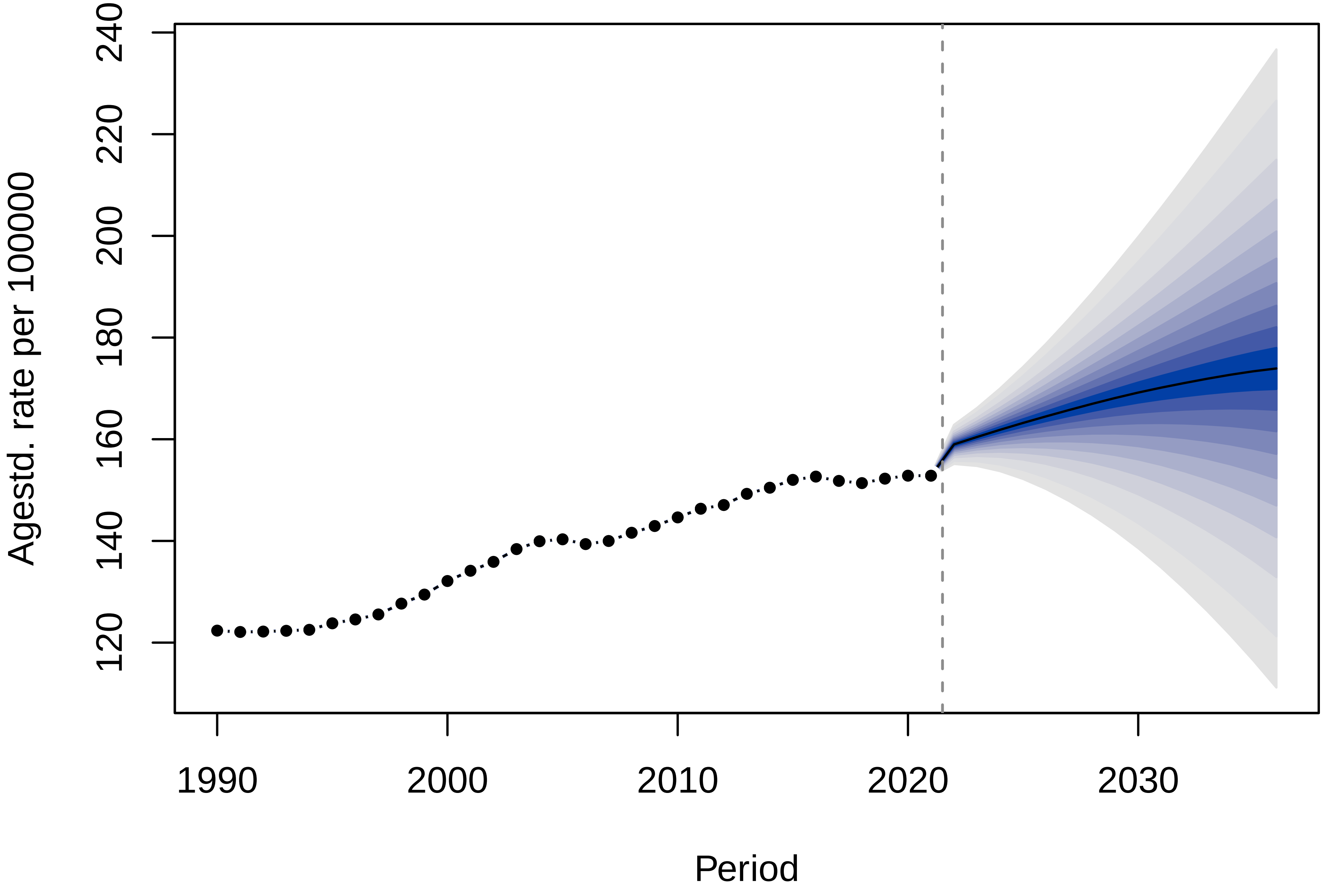

Agestd. rate per 100000
120
140
160
180
200
220
240
1990
2000
2010
2020
2030
Period

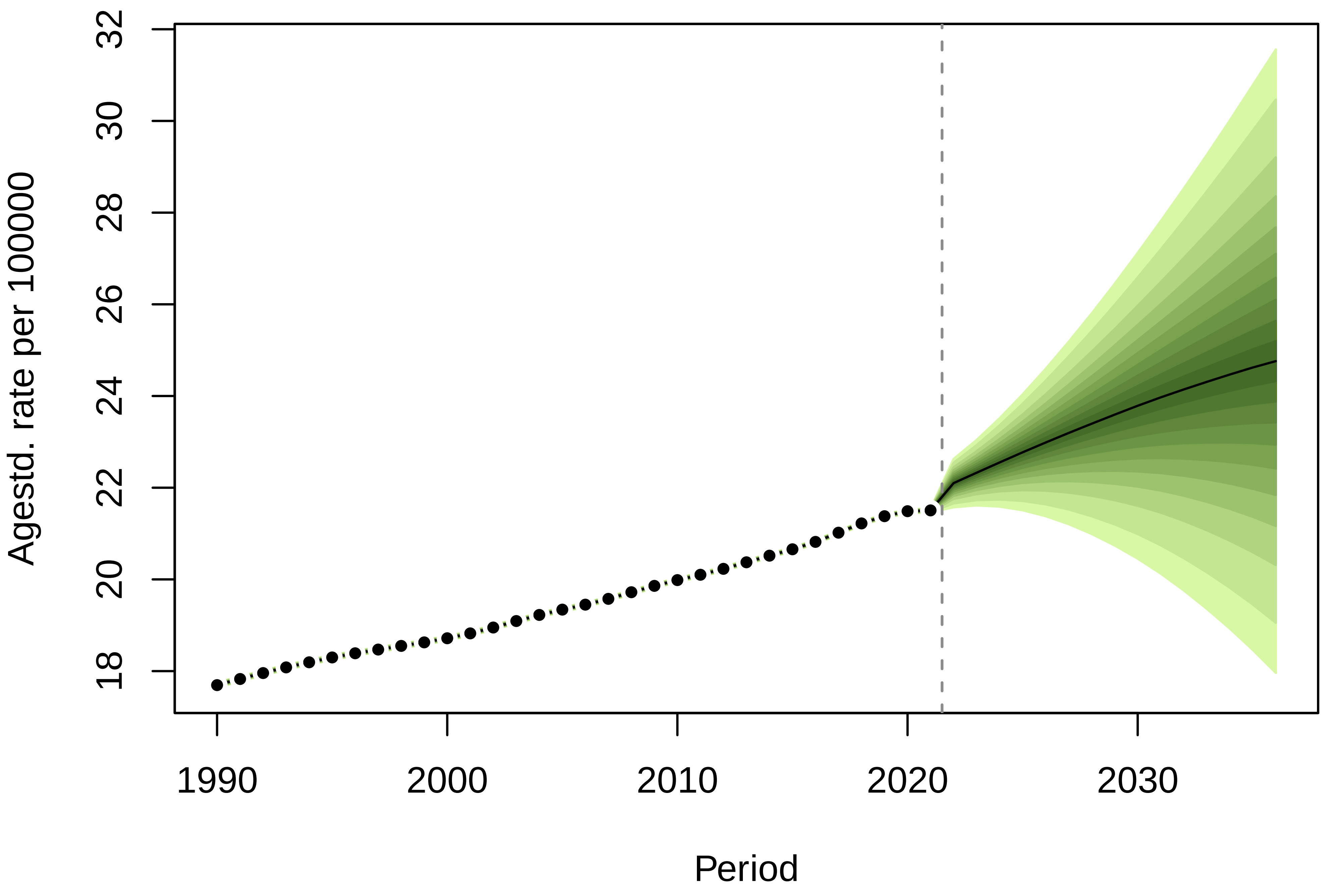

Agestd. rate per 100000
18
20
22
24
26
28
30
32
1990
2000
2010
2020
2030
Period

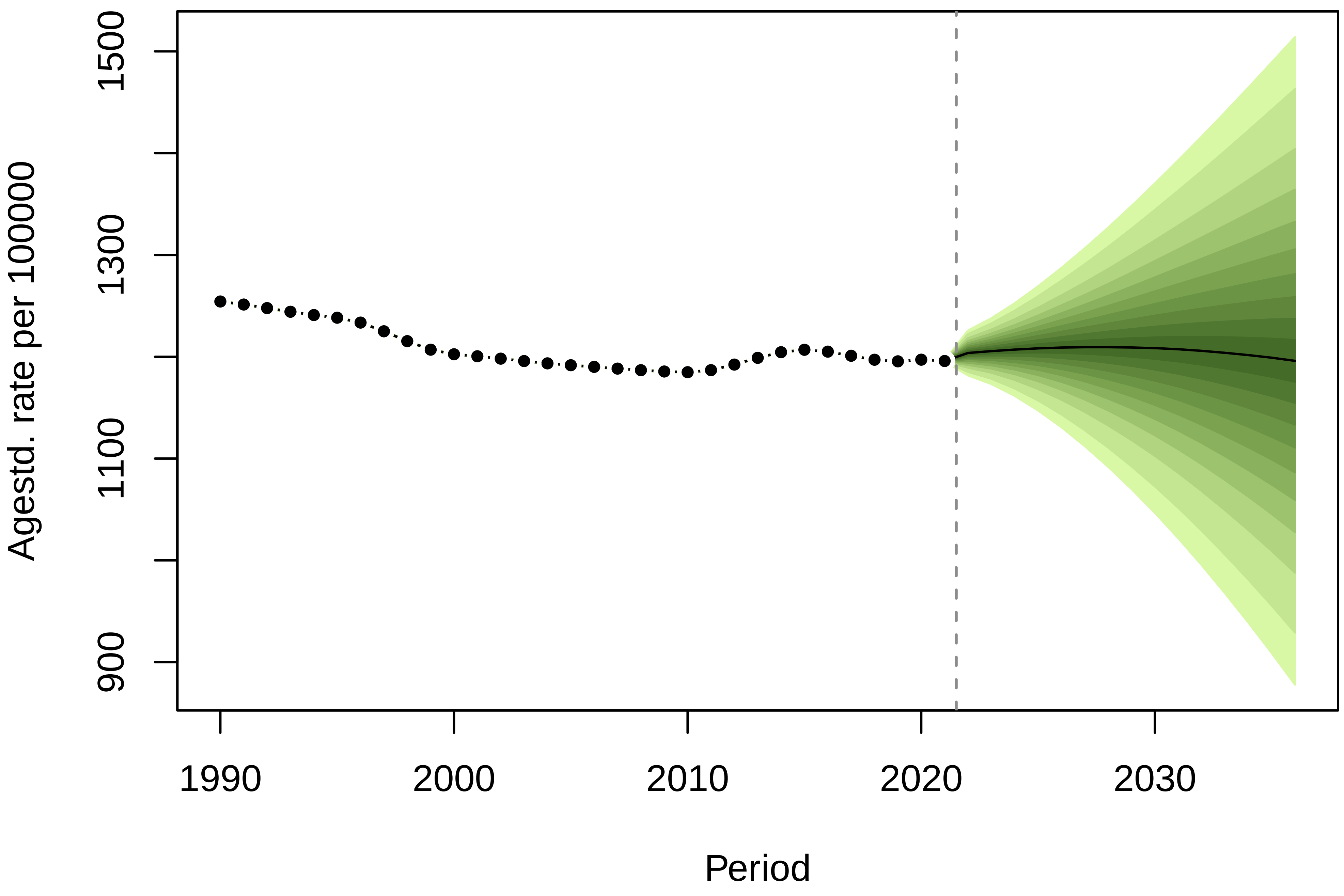

Agestd. rate per 1000000
900
1100
1300
1500
1990
2000
2010
2020
2030
Period

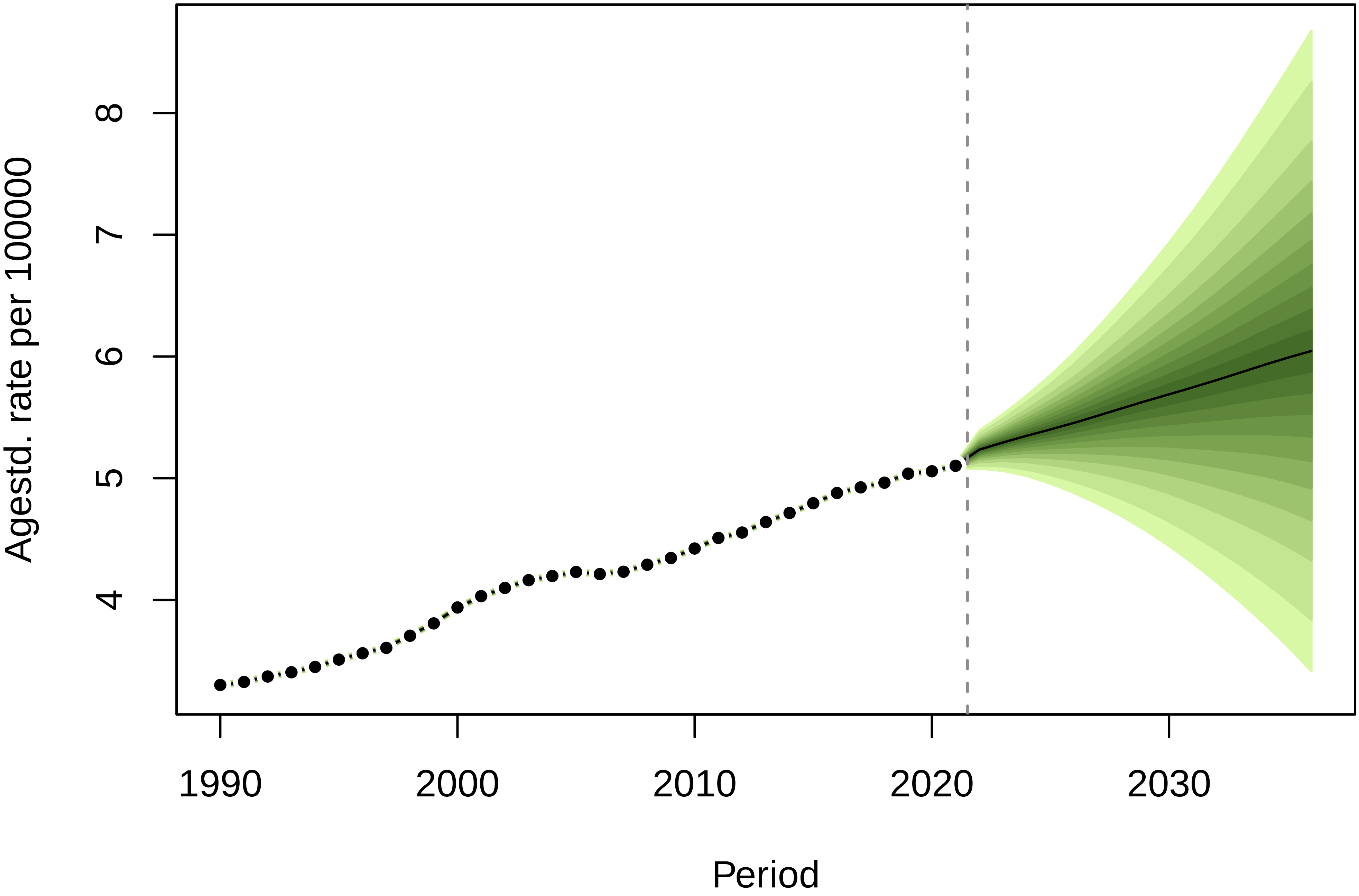

Agestd. rate per 1000000
4
5
6
7
8
1990
2000
2010
2020
2030
Period

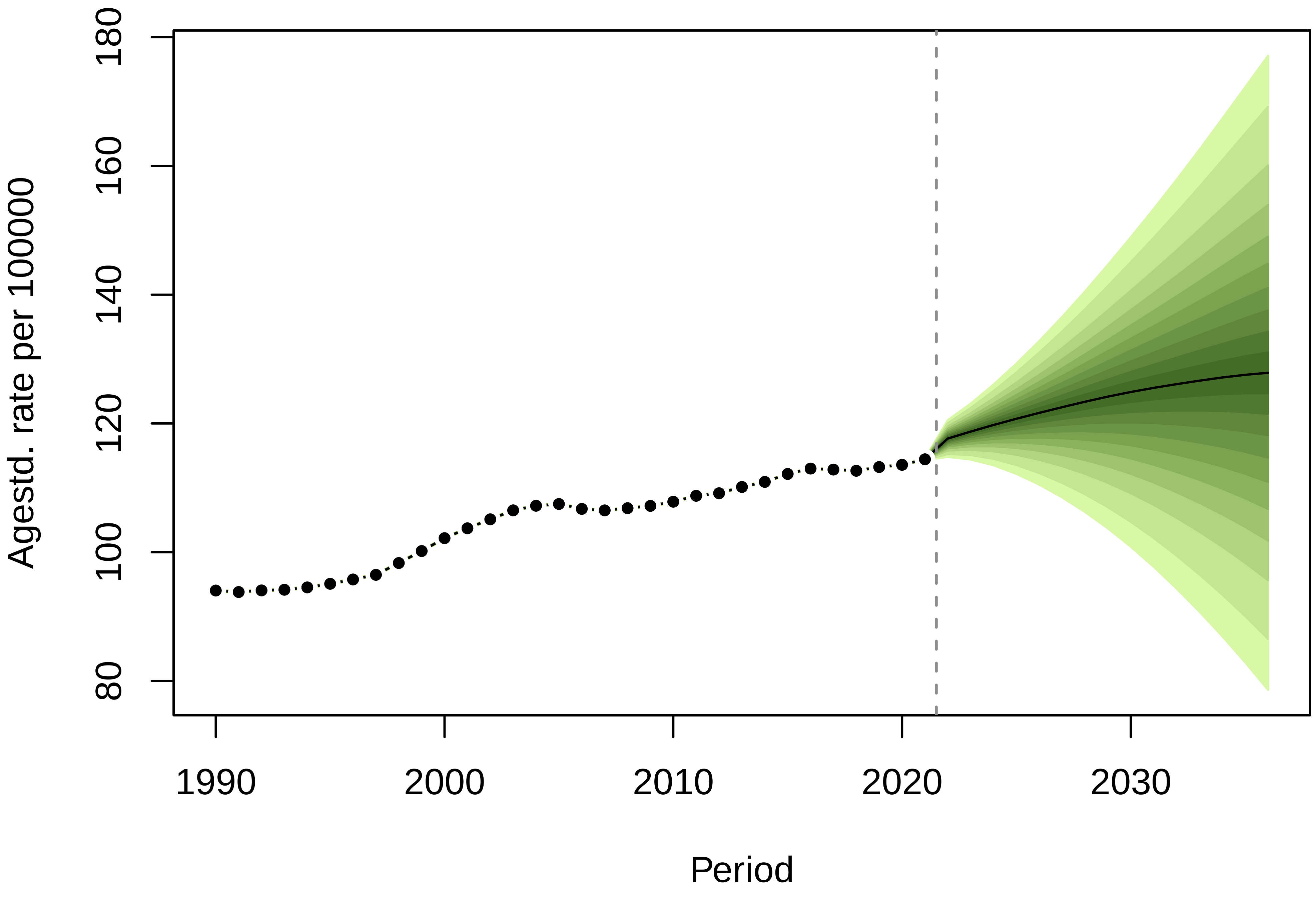

Agestd. rate per 1000000
180
160
140
120
100
80
1990
2000
2010
2020
2030
Period

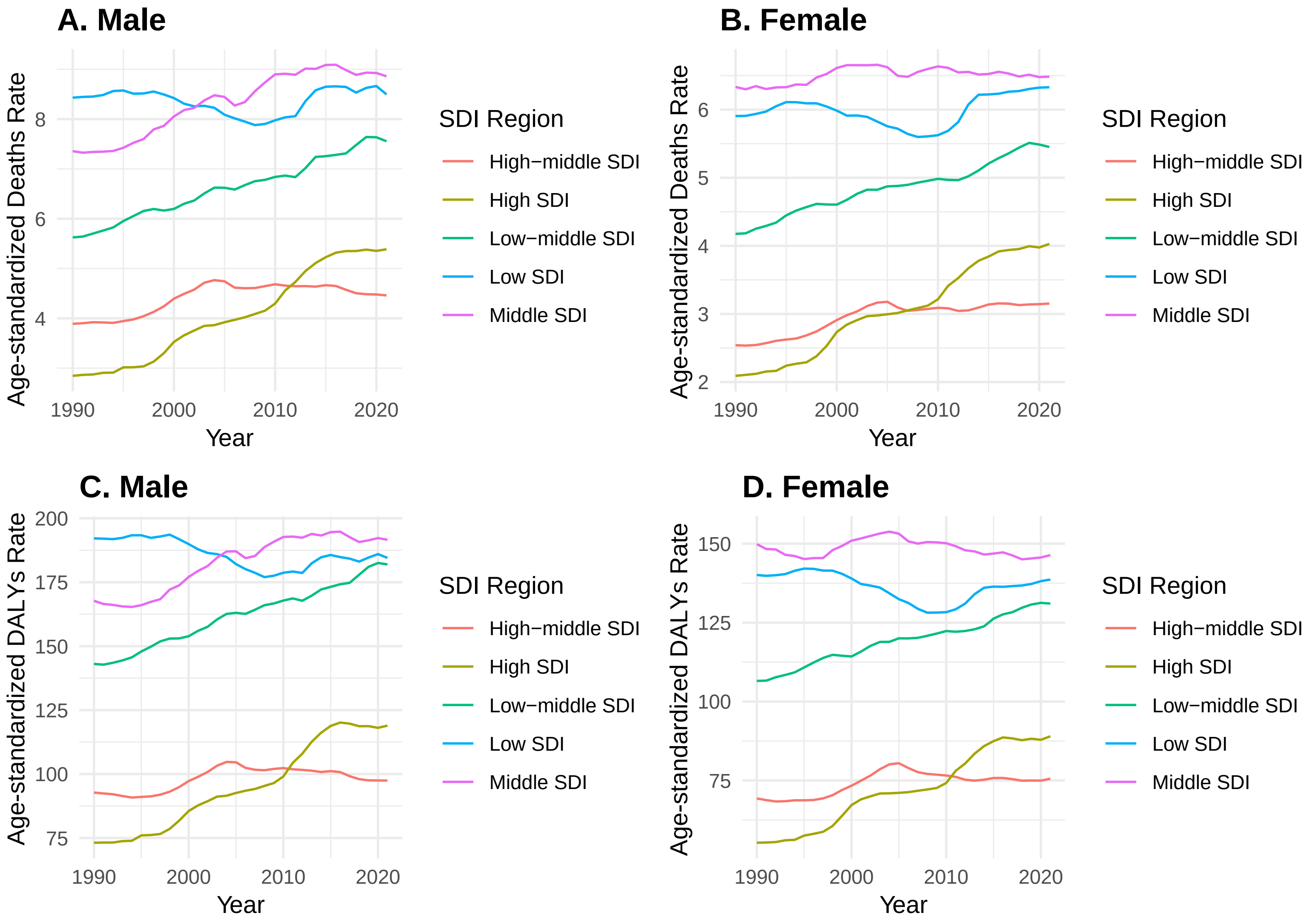
A. Male
B. Female
C. Male
D. Female
Age-standardized Deaths Rate
Age-standardized DALYs Rate
Year
1990
2000
2010
2020
8
6
4
5
3
2
200
175
150
125
100
75
SDI Region
High−middle SDI
High SDI
Low−middle SDI
Low SDI
Middle SDI

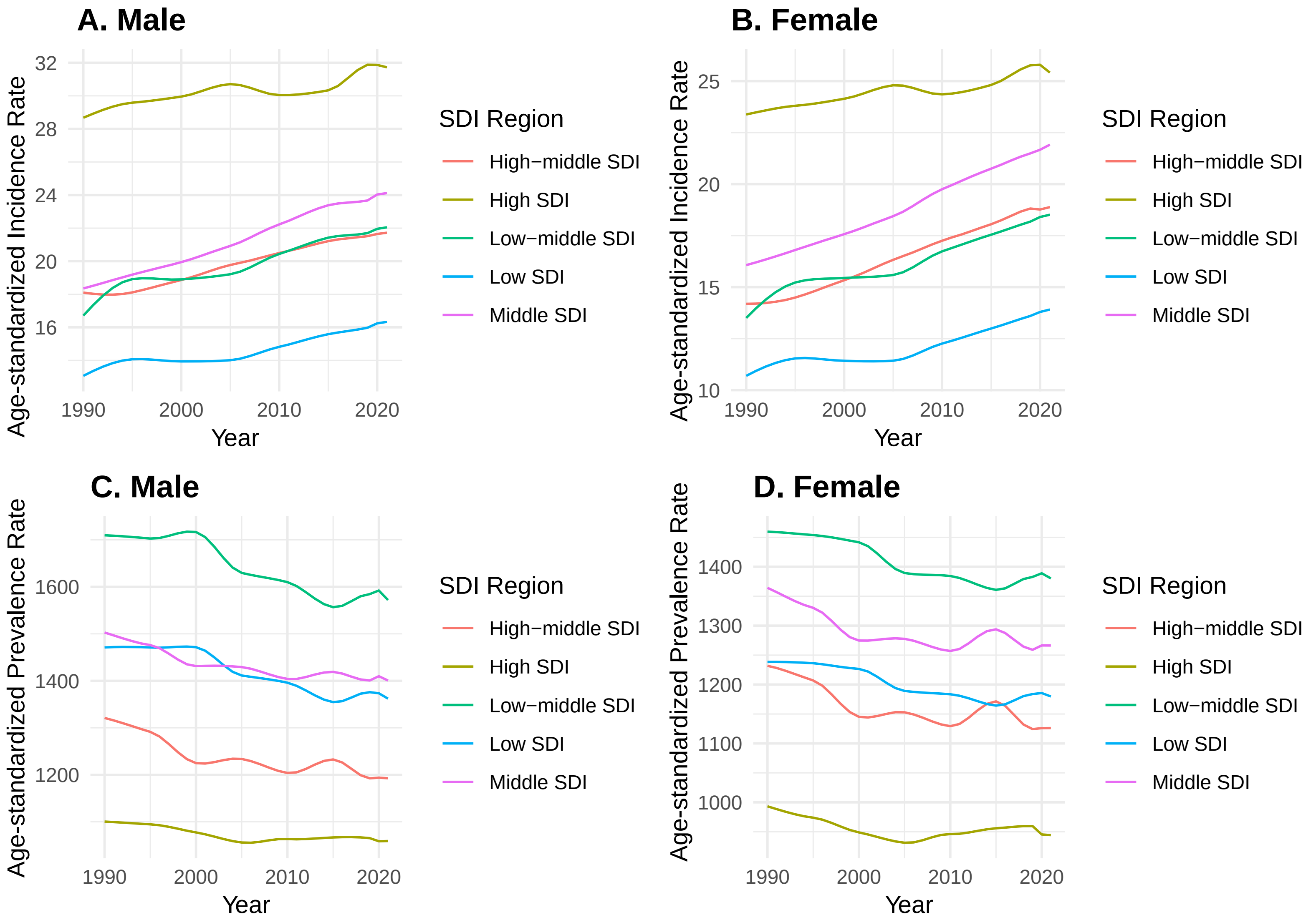
A. Male
B. Female
C. Male
D. Female
Age-standardized Incidence Rate
Age-standardized Prevalence Rate
Year
1990
2000
2010
2020
32
28
24
20
16
25
15
10
1600
1400
1200
1300
1100
1000
SDI Region
High−middle SDI
High SDI
Low−middle SDI
Low SDI
Middle SDI
